# A Review on Applications of Time-Lapse Electrical Resistivity Tomography Over the Last 30 Years : Perspectives for Mining Waste Monitoring

**DOI:** 10.1007/s10712-022-09731-2

**Published:** 2022-08-12

**Authors:** Adrien Dimech, LiZhen Cheng, Michel Chouteau, Jonathan Chambers, Sebastian Uhlemann, Paul Wilkinson, Philip Meldrum, Benjamin Mary, Gabriel Fabien-Ouellet, Anne Isabelle

**Affiliations:** 1 Université du Québec en Abitibi-Témiscamingue (UQAT), Rouyn-Noranda, Québec J9X 5E4 Canada; 2 Polytechnique Montréal, Montréal, Québec H3T 1J4 Canada; 3 Research Institute of Mines and Environment (RIME), Québec, Canada; 4 British Geological Survey (BGS), Environmental Science Centre, Nottingham, NG12 5GG United Kingdom; 5 Lawrence Berkeley National Laboratory (LBNL), Berkeley, California 94720 United States; 6 Department of Geosciences, University of Padua, Padua, 35122 Italy

**Keywords:** Time-lapse electrical resistivity tomography, Mining wastes monitoring, Geotechnical and geochemical stability, Remote autonomous monitoring, Early warning systems

## Abstract

Mining operations generate large amounts of wastes which are usually stored into large-scale storage facilities which pose major environmental concerns and must be properly monitored to manage the risk of catastrophic failures and also to control the generation of contaminated mine drainage. In this context, non-invasive monitoring techniques such as time-lapse electrical resistivity tomography (TL-ERT) are promising since they provide large-scale subsurface information that complements surface observations (walkover, aerial photogrammetry or remote sensing) and traditional monitoring tools, which often sample a tiny proportion of the mining waste storage facilities. The purposes of this review are as follows: (i) to understand the current state of research on TL-ERT for various applications; (ii) to create a reference library for future research on TL-ERT and geoelectrical monitoring mining waste; and (iii) to identify promising areas of development and future research needs on this issue according to our experience. This review describes the theoretical basis of geoelectrical monitoring and provides an overview of TL-ERT applications and developments over the last 30 years from a database of over 650 case studies, not limited to mining operations (e.g., landslide, permafrost). In particular, the review focuses on the applications of ERT for mining waste characterization and monitoring and a database of 150 case studies is used to identify promising applications for long-term autonomous geoelectrical monitoring of the geotechnical and geochemical stability of mining wastes. Potential challenges that could emerge from a broader adoption of TL-ERT monitoring for mining wastes are discussed. The review also considers recent advances in instrumentation, data acquisition, processing and interpretation for long-term monitoring and draws future research perspectives and promising avenues which could help improve the design and accuracy of future geoelectric monitoring programs in mining wastes.

## Introduction

**Fig. 1:**
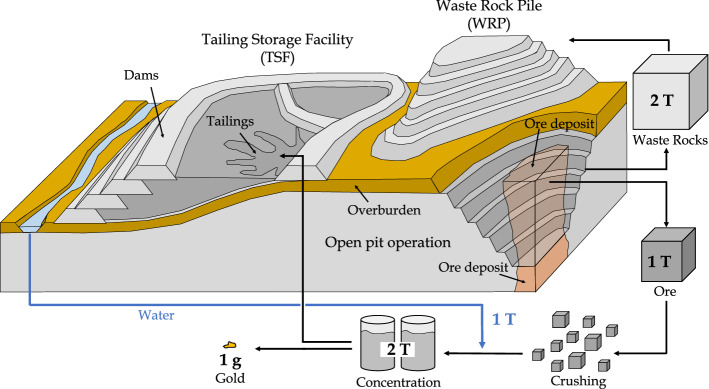
Diagram of an open-pit mine operation and simplified mass balance of wastes and minerals

Large volumes of wastes are produced as a result of mining and overburden removal, and during the processing of ore (Nassar et al. 2022). Figure 1 presents a simplified mass balance of mining wastes for a gold open-pit operation with an average grade of 1 g/ton. In this example, two tons of waste rocks are extracted from the open pit and stored into waste rock piles (WRP) in order to reach and extract one ton of ore. The ore is then crushed into fine particles, mixed with one ton of water to concentrate and extract one gram of gold. The remaining two tons of crushed rocks and water are called tailings and are generally pumped into tailing storage facilities (TSF) (Bussière and Guittonny 2020a). Although other types of mining operations generate less mining wastes (e.g., underground mining), this example illustrates that for most mineral deposits, more than $99~\%$ of the rocks extracted from large-scale and low-grade open-pit operations would be stored either in WRPs or TSFs (Vriens et al. 2020a).

From a global point of view, it is estimated that between 29 000 and 35 000 active and inactive TSFs contain over 200 billion tons of tailings worldwide (World Mine Tailings Failures database, WMTF). In Canada alone, the Global Tailings Portal (GTP) program initiated in 2019 reports more than 200 TSFs which contain approximately 4.5 billion tons of tailings (data accessed on October 2021). Although these programs provide for the first time free access to searchable databases of mining wastes worldwide, they might not include all mining sites (e.g., orphaned mines or unregistered active mines) and do not provide information concerning the surface area occupied by mining wastes, which is a key aspect for the assessment of mining operations environmental legacy (Bussière et al. 2020).

**Fig. 2:**
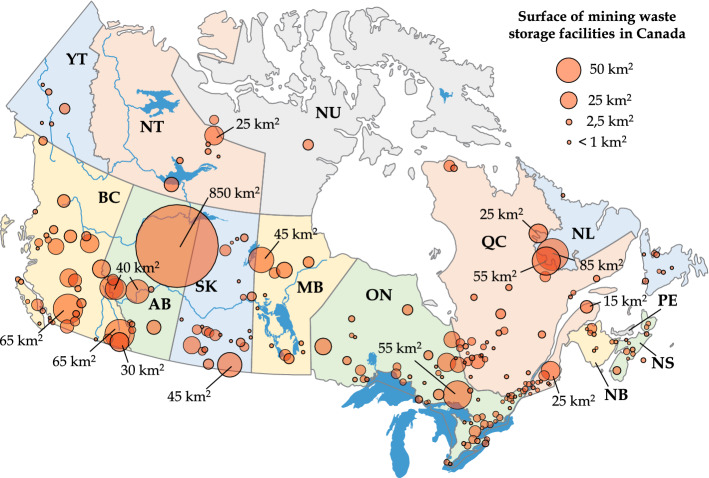
Distribution and surface of mining waste storage facilities across Canada in 2020 Each mining site in Canada (active, closed or abandoned) has been identified and the areas occupied by TSFs, WRPs and open-pits have been calculated with Google Earth satellite imagery. The database and interactive maps can be accessed and downloaded through https://adridim.github.io/review2022/0_welcome.html

We have compiled a specific database to address these limitations and estimate the surface of mining wastes in Canada with Google Earth satellite imagery. Figure 2 shows the distribution of most Canadian mining operations (both active and inactive), and the estimated surface of TSFs, WRPs and open-pits. The mining sites have been identified from the GTP and several other sources including Canadian National Pollutant Release Inventory (NPRI), United States Geological Survey (USGS) and International Commission on Large Dams (ICOLD) databases. In total, the 200 TSFs, 185 WRPs and 200 open-pits identified across Canada cover approximately 1 500 km$^{2}$, 600 km$^{2}$ and 500 km$^{2}$, respectively. These mining waste storage facilities can reach large dimensions (70 TSFs exceed 100 m in height worldwide) (Kossoff et al. 2014), cover large surfaces (20 TSFs and 15 WRPs exceed 10 km$^{2}$ in Canada) (Bussière and Guittonny 2020a) and have even been described as “the largest man-made structures on earth” by some authors (Bowker and Chambers 2015; Lyu et al. 2019b; Owen et al. 2020).

Although increasing efforts are being made toward the recycling of non-renewable mineral resources, the growing need for base and precious metals from both developed and developing countries as well as the energy transition requires the development of existing mining operations and the exploitation of new deposits (Bussière and Guittonny 2020a; Vriens et al. 2020a). However, a global depletion of ore grades has been observed worldwide for various metals over the last ten years since most high-grade deposits have already been mined out (Calvo et al. 2016; Mudd 2007). In the meantime, recent technological advances in milling and concentration processes have decreased the cost of large-scale mining operations (Bussière and Guittonny 2020a; Rötzer and Schmidt 2018). As a result, the global trend is toward larger-scale and lower-grade deposit exploitation, which increases the generation of mining wastes (Chambers 2016; Kossoff et al. 2014). In practice, 40 to 50 billion tons of tailings are expected to be generated over the next five years, which would represent an increase of nearly 25 % of the current TSFs capacity worldwide by 2025 (WMTF) and a similar increase could be expected for WRPs.

As reported by Bussière (2007), “the safe disposal of [mining wastes] is certainly one of the most important environmental challenges for the mining industry.” This can be explained by two major aspects; the physical and the geochemical stability of both tailings and waste rocks. From the geotechnical perspective, the poor mechanical properties of tailings (fine particles initially saturated) increase the risk of TSF failures if the containment dams are poorly designed and/or exposed to extreme conditions (earthquakes, landslides or heavy rains) (Azam and Li 2010; Bowker and Chambers 2015). In recent years, several catastrophic TSF failures have been reported worldwide, claiming hundreds of lives, displacing thousands of families and releasing hundreds of million tons of tailings into the environment, with an average estimated cost of over $\$500$ million USD per failure (Clarkson et al. 2021; Rotta et al. 2020). From the geochemical perspective, the generation of contaminated drainage from mining wastes has been recognized as a potential threat to the environment since Georgius Agricola’s book in 1556 entitled *De re metallica* (Agricola and Hoover 1556/1912), and has been intensively studied worldwide since the 1970s (e.g., Blowes et al. (2003); Nordstrom et al. (2015)), especially in recent years (Aznar-Sánchez et al. 2018; Tomiyama and Igarashi 2022). Acid mine drainage (AMD) is generally caused by the oxidation of sulfides (e.g., pyrite or pyrrhotite) present in the waste rocks or tailings when the mining wastes are exposed to water and oxygen flows from the atmosphere (Plante et al. 2020). Sulfide oxidation generates acidity, and thus decreases pH below 7 to extremely low values (even negative), which in turn increases the solubility of most metal species (e.g., Nordstrom et al. (2000)). When the oxidation process is poorly controlled, AMD can have a significant impact on groundwater quality in nearby ecosystems and may extend to distances if acidic waters discharge into water streams (Blowes et al. 2003; Rezaie and Anderson 2020). AMD can been responsible for making both surface and groundwater unsuitable for the use of plants, animals and humans, causing diseases and disorders (e.g., Rotta et al. (2020)).

As discussed by Wilson (2021), the recent TSF failures have generated an unprecedented response in the global mining community, from shareholders, stakeholders to governmental legislation including public opinion. As a result, more efforts have been directed toward innovative construction designs that would increase TSF geotechnical stability such as the inclusion of waste rocks within TSFs (James et al. 2013) or the use of dry tailings to favor unsaturated conditions and reduce the risk of liquefaction (Bussière 2007; Wilson 2021). In the meantime, different reclamation approaches have been proposed to reduce the economic and environmental burden that AMD-generating TSFs and WRPs represent for communities, governments and taxpayers (Bussière and Guittonny 2020a; Madejón et al. 2021). Among other techniques, the construction of multi-layer covers on WRPs or TSFs to reduce water and/or oxygen flows toward the reactive mining wastes is particularly promising. Indeed, such reclamation approaches allow controlling at the source the long-term generation of contaminated drainage and to reuse mining wastes (Park et al. 2019; Vriens et al. 2020a).

Although “conservative design and diligent operation” of TSFs and WRPs are critical for ensuring both geotechnical and geochemical long-term stability, Hui et al. (2018) stressed the need for “continuous, efficient and cost-effective monitoring” of these large-scale structures, before and after mine closure. As discussed by Clarkson and Williams (2020), real-time monitoring of TSF geotechnical stability allows for a better characterization of potential internal deformations, water table elevation in the tailings as well as abnormal seepage. Such monitoring enables TSFs operators to take the possible measures to mitigate the consequence or even interrupt the deterioration before failure occurs (Clarkson et al. 2021; Hui et al. 2018). From the geochemical stability perspective, three types of processes can diminish the long-term performance of multi-layer covers according to the MEND report 2.21.4d (2004). The report identified physical processes (e.g., erosion, extreme precipitations or droughts or freeze/thaw cycles), chemical processes (e.g., mineralogical dissolution or consolidation) and biological processes (e.g., root penetration, root water uptake, burrowing animals or human intervention) (MEND 2004), all of which can occur both locally or globally, progressively or suddenly (Rykaart et al. 2006). Here again, Bussière et al. (2020) highlighted the need for long-term monitoring of covers for TSFs and WRPs reclamation since a decrease in performance could be detected by a drop or an increase in water content, suction or temperature for instance, which would allow to take actions in the early stages of the tailings oxidation process (Dagenais et al. 2001). As noted by the MEND report, there is no clear consensus or legislation regarding the duration of such monitoring programs. However, some mining companies have developed closure standards which specify that the geotechnical and geochemical stability of mining waste storage must be ensured for a 200 to 500 year time frame (Bussière and Guittonny 2020b; MEND 2004).

As reported by Hui et al. (2018), the conventional monitoring techniques used to assess the stability of WRPs and TSFs can be classified into two categories; surface observations and point sensing (Hui et al. 2018). Surface observations provide detailed information over large-scales, but only skin deep (e.g., visual inspections, satellite imagery for moisture content and aerial photogrammetry, interferometric synthetic aperture radar—InSAR, or light detection and ranging—LIDAR, for surface deformations) (Clarkson and Williams 2020; Smethurst et al. 2017). On the contrary, point sensing instruments are generally installed within TSFs and WRPs to monitor a physical property, but only with a limited volume of investigation, typically of a few centimeters around each sensor (e.g., geodetic systems for ground displacement, piezometers for pore-water pressure, hydrogeological sensors for moisture content, suction or temperature) (Bussière et al. 2020; Hui et al. 2018). Generally, several monitoring stations (e.g., using vertical profiles of point sensors) are placed at critical locations to monitor the stability of WRPs or TSFs under various conditions (Bussière et al. 2020; MEND 2004). Although there is no single rule for the number of instruments needed, it is generally established that a dense network of instruments is needed to cover a representative area of mining waste storage facilities (MEND 2004). As discussed by Bussière et al. (2020) and Rykaart et al. (2006) such a monitoring approach can represent significant costs given the spatial extent of TSFs and WRPs.

The recent reviews from Loke et al. (2013), Binley et al. (2015) and Slater and Binley (2021) have highlighted the emergence of time-lapse electrical resistivity tomography (TL-ERT) as a promising technique for monitoring of various subsurface processes across multiple scales. This non-destructive imaging approach has been combined with surface observations and point sensors measurements for long-term monitoring of landslides (Whiteley et al. 2019), permafrost (Mollaret et al. 2019), infrastructure (Chambers et al. 2014) and many other fields (Falzone et al. 2019). Although several other geophysical methods such as self-potential (Jougnot et al. 2015; Soupios and Kokinou 2016), induced polarization (Abdulsamad et al. 2019; Saneiyan et al. 2019), active and passive seismic (Grandjean et al. 2009; Olivier et al. 2017) or ground-penetrating radar (Giertzuch et al. 2021; Steelman et al. 2017) have been applied in similar contexts, the focus of this review is on TL-ERT since this technique is cost-efficient, robust and readily deployable for large-scale monitoring. Furthermore, TL-ERT is one of the most well understood near surface geophysical techniques, and is particularly sensitive to moisture driven processes, which play a key role in mining waste stability.

In the context of mining wastes monitoring, TL-ERT could be applied as a complementary method to extend traditional measurements, thus reducing the number of conventional sensors needed for large-scale TSfs and WRPs while increasing the volume of investigation for long-term stability monitoring programs (Bussière et al. 2020). Although several examples have been reported in the literature (e.g., Dimech et al. (2019); Tresoldi et al. (2020a)), the relative scarcity of studies using TL-ERT for mining wastes monitoring is surprising given (i) the critical need for efficient long-term and large-scale monitoring techniques in WRPs and TSFs, (ii) the numerous applications of static ERT imaging in this domain reported by the review from Martinez-Pagan et al. (2021), and (iii) the recent developments which have improved TL-ERT for long-term remote monitoring of various subsurface processes (Binley and Slater 2020; Slater and Binley 2021).

In this regard, the present review summarizes the state of the art and the development of time-lapse ERT over the last 30 years. A database of TL-ERT studies since 1990 is used to identify and describe the different types of application, and review the recent developments that made TL-ERT a recognized and complementary tool for long-term remote monitoring. In the meantime, a database of studies using ERT for mining waste characterization and monitoring allows to identify promising avenues for long-term monitoring of TSFs and WRPs. The article reviews some lessons learned from three decades of TL-ERT development in other domains. Finally, suggestions are proposed to overcome the challenges that could arise from a more widespread application of TL-ERT for long-term mining waste monitoring. In particular, several research perspectives are suggested to improve the accuracy of future ERT monitoring programs and upscale stability assessment in mining waste storage facilities.

## Monitoring Subsurface Processes With Time-Lapse Electrical Resistivity Tomography

### Objectives of TL-ERT

Electrical conductivity (EC in S/m) (or its inverse electrical resistivity ER in $\Omega$m) describes the ability of a medium to conduct electrical current under a given electrical field (Samouëlian et al. 2005). Three electrical conduction modes can contribute to the porous medium EC (Corwin and Scudiero 2019; Revil et al. 2012):

- **Electrolytic conduction** is caused by the displacement of ions present in the interstitial fluid (Revil et al. 2014) and is a major contribution for ionized fluids (e.g., saline water). It can be negligible for low-porosity media, low ion content fluids (e.g., pure water) or frozen fluids.
- **Surface conduction** occurs at the surface of solid grains due to the accumulation of ions in the electrical double layer surrounding each grain. Surface conduction can be a major contribution to the total conduction when the medium contains clay particles (Revil et al. 2012).
- **Solid matrix conduction** is caused by the displacement of mobile electrons within solid grains and is negligible for most rocks and soils which do not contain metallic particles (Rhoades et al. 1989).

Since the electrical conductivity of a medium depends on many parameters (e.g., water content, temperature and ionic content of the interstitial fluid), it can be used as a proxy to image various subsurface processes (Falzone et al. 2019). Several reviews present the physical parameters affecting porous medium EC such as Friedman (2005) and Samouëlian et al. (2005). Corwin and Scudiero (2019) also reviewed many published studies using EC to image (a) water content, (b) pore fluid composition (salinity, ion content and pH), (c) solid matrix properties (grain size, mineralogy and compaction), (d) temperature and (e) organic materials. Moreover, they provided a review of the relationships found in the literature to link EC with these key parameters (referred to as petrophysical relationships).

Arguably, the semi-empirical Archie’s law (1942) (Archie et al. 1942) is the most commonly used petrophysical relationship to link electrolytic conduction $\sigma _{\mathrm {el}}$, pore fluid EC $\sigma _\mathrm {w}$, saturation $S_\mathrm {w}$ and porosity $\phi$:

1 $$\begin{aligned} \sigma _{\mathrm {el}} = \sigma _\mathrm {w} \cdot \phi ^{m} \cdot S_\mathrm {w}^n \end{aligned}$$

where *m* and *n* are, respectively, the cementation and the saturation exponents, related to the pore structure, tortuosity, connectivity and fluid properties (Glover 2009). Other popular models based on Archie’s law also incorporate surface conduction and solid matrix conduction such as Waxman-Smits relationship (1968) (Waxman and Smits 1968) or the Generalized Archie’s Law (Glover 2010). Notably, the latter has been applied to estimate moisture content in mine tailings from bulk EC (Canales et al. 2020).

ERT allows imaging of the ER distribution of the subsurface with electrodes inserted in the ground. Two electrodes are used to transmit current and two potential electrodes measure the resultant voltage, which depends on the subsurface resistivity distribution (Lesmes and Friedman 2005). Many measurements are made using different combinations of current and potential bipoles. The resulting data set is then inverted to recover a distribution of electrical resistivity (see Sect. 2.3 for details on ERT inversion). At the end of the inversion procedure, the resistivity distribution obtained is consistent with the measured data. This distribution is assumed to be representative of the true subsurface resistivity distribution, subject to limitations of a priori assumptions, data and modeling errors, model resolution and non-uniqueness (Whiteley et al. 2019).

As reported by Binley and Slater (2020), the first application of resistivity measurements for subsurface investigation dates back to Conrad Schlumberger’s work in the 1910s (Schlumberger 1920). Since then, resistivity measurements have been used to recover (i) one-dimensional (1D) vertical electrical soundings with four electrodes using different spacing in the 1920s, (ii) two-dimensional (2D) profiles of resistivity using linear arrays of electrodes installed at the surface in the 1960s and (iii) three-dimensional (3D) images using several parallel linear arrays of surface or borehole electrodes in the 1970s (Binley and Slater 2020) and more complex 3D arrays since the 2000s (e.g., Star array (Clément et al. 2010; Rucker 2015) and L-array (Tejero-Andrade et al. 2015)). Since the 1990s, subsurface monitoring has been conducted by time-repetitive ERT imaging at the same location (Singha et al. 2015). This approach, referred to as TL-ERT, is used to recover spatio-temporal changes in medium ER, and to monitor various dynamic processes such as tracer migration (Perri et al. 2012), water infiltration (Hübner et al. 2017), root water uptake (Mary et al. 2020), permafrost dynamic (Uhlemann et al. 2021) or geothermal operations (Robert et al. 2019).

**Fig. 3:**
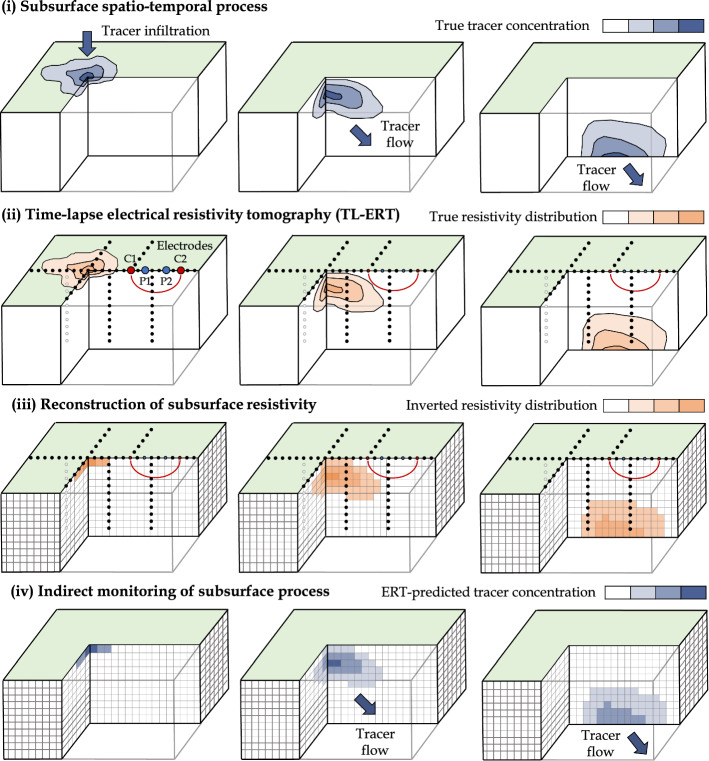
Example of TL-ERT monitoring of a tracer infiltration with surface and borehole electrodes. (i) Top panel shows the true spatio-temporal distribution of tracer concentration in the medium, (ii) medium panel presents the corresponding distribution of electrical resistivity and (iii) the inverted distribution of resistivity obtained from TL-ERT monitoring. Finally, (iv) bottom panel shows the ERT-predicted tracer concentration using petrophysical relationship (based on Singha et al. (2015))

Figure 3 illustrates a TL-ERT survey used to monitor tracer flow in a medium (based on Singha et al. (2015)). The first panel represents the initial tracer distribution. Assuming that the tracer resistivity is different from the initial pore fluid resistivity, the evolution of tracer concentration causes changes in the medium resistivity as shown on the second panel. Several ERT snapshots can then be obtained with surface and/or borehole electrodes remaining at the same position during a period of time. The third panel illustrates the time-lapse inversion results : the medium resistivity distribution is reconstructed for a discretized medium at the measured time steps. Finally, the tracer concentration can be estimated from the inverted resistivity distribution using a petrophysical relationship such as Archie’s law (Equation 1) assuming that pore fluid resistivity is the only parameter changing over time and that there is no matrix or mineral surface conductance (fourth panel).

### Key Parameters of TL-ERT Surveys

**Fig. 4:**
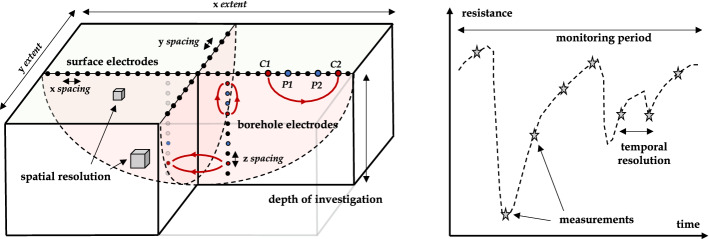
Spatio-temporal parameters of TL-ERT surveys (inspired from Rucker (2014))

Each TL-ERT monitoring survey can be described by several key parameters which are divided into two categories: the spatial and the temporal parameters illustrated in Fig.  4. As discussed by several authors (e.g., Rucker (2014); Tildy et al. (2017)), such parameters should be appropriately determined for each specific field site objectives as well as for each subsurface process that is to be monitored. The spatial parameters on the left part of Fig. 4 include (i) the spatial extent, (ii) the depth of investigation and (iii) the spatial resolution. The temporal parameters on the right part of Fig. 4 are (i) the monitoring period and (ii) the temporal resolution (Friedel 2003). Following the example of Rucker (2014), a brief description of each of these spatio-temporal parameters is proposed below.

- **Spatial extent** describes the dimension of the investigated area (*x* and *y* extent) and is determined by electrode positions (i.e., geometry of the electrode layout, number of electrodes and *x*, *y* spacing). The spatial extent of a TL-survey can range from a few centimeters for laboratory experiments (Corona-Lopez et al. 2019) to hundreds of meters for large-scale surveys (Uhlemann et al. 2017).
- **Spatial resolution** refers to the dimension of the smallest resistivity anomaly that can be imaged by ERT. Although explicitly calculated by some studies (e.g., Friedel (2003)), spatial resolution is generally assessed with sensitivity distribution and depends on electrode spacing, electrode positioning (e.g., surface or borehole electrodes (Singha et al. 2015)) and measurement protocols (Stummer et al. 2004).
- **Depth of investigation (DOI)** corresponds to the depth below which changes of resistivity in the medium would not affect the measurements (Oldenburg and Li 1999) (i.e., *z* extent of the survey). DOI is determined by spatial extension and measurement protocols (Samouëlian et al. 2005) and can be either explicitly calculated (Oldenburg and Li 1999), assessed from sensitivity distribution (Carey et al. 2017) or from basic rules of thumbs (Greggio et al. 2018; Henderson et al. 2010).
- **Monitoring period** represents the duration of the time-lapse survey (i.e., time difference between the first and the last ERT snapshot). Monitoring periods found in the literature vary from a few hours for short surveys (Kuras et al. 2009), a few months for seasonal dynamics (Jodry et al. 2019; Mojica et al. 2013) to several years for long-term studies (Caterina et al. 2017; Palis et al. 2017) (e.g., more than 20 years for permafrost monitoring (Mollaret et al. 2019)).
- **Temporal resolution** refers to the fastest dynamic event that can be reconstructed by ERT (i.e., temporal counterpart of spatial resolution). Temporal resolution depends on the ERT measurement rate (e.g., one snapshot per day (Palis et al. 2017)) and the time needed to perform each ERT snapshot. Temporal resolution should be defined appropriately for each TL-ERT survey to avoid motion blur if the process is occurring at a significantly faster rate than the ERT measurement frequency (e.g., quick tracer infiltration (Kuras et al. 2009)).
- **Measurement protocol** corresponds to the ensemble of four electrode measurements used to measure each ERT snapshot. Given the large number of possible configurations (Loke et al. 2013), the measurement should be designed to provide the best trade-off between how many measurements are made for an ERT snapshot (i.e., spatial resolution) and how much time it takes (i.e., temporal resolution) (Wilkinson et al. 2012).

As discussed by Whiteley et al. (2019), the monitoring strategy is another important parameter describing each TL-ERT survey. Three categories of strategies can be identified in the literature depending on the duration of TL-ERT as well as the type of measurements carried out:

- **Transient measurements** typically involve static ERT measurements repeated after a certain period of time (e.g., one month to one year) to characterize seasonal variations. The measuring devices (cables, electrodes, resistivity meters) are usually installed during a few hours for each snapshot and removed from the field after each acquisition (e.g., Beff et al. (2013); Binley et al. (2015)).
- **Controlled tests** are usually short monitoring campaigns with high temporal resolution (e.g., one image every hour) which aim to image the medium in response to artificial perturbations (e.g., irrigation (Dimech et al. 2019; Hardie et al. 2018), water depletion due to pumping (Kuras et al. 2009), injection of tracer (Monego et al. 2010)) or natural perturbations (e.g., rain events (Scaini et al. 2017)) .
- **“Semi”-permanent installations** typically use a dedicated measurement system permanently installed on the site during long periods of time (e.g., 1 year or more) with high temporal resolution (e.g., one ERT snapshot per day) (see for instance Chambers et al. (2014); Mollaret et al. (2019)). This type of installation is usually preferred to monitor long-term and/or slow-changing subsurface processes (Slater and Binley 2021).

### TL-ERT Data Acquisition, Processing and Inversion

**Fig. 5:**
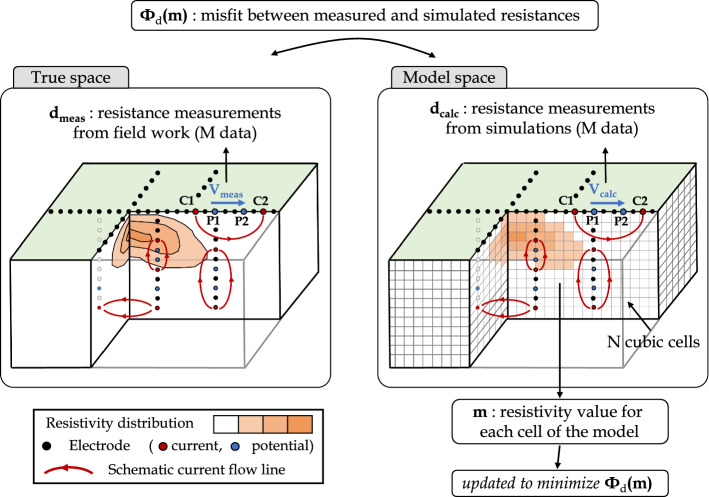
Diagram of ERT inversion routine used to reconstruct distribution of electrical resistivity $\rho$

Figure 5 synthesizes graphically ERT field measurements, data filtering and inversion, which are the three main steps used to reconstruct the subsurface distribution of ER at a specific time (i.e., static imaging). Each of these steps is discussed below :

(i) In the field, each measurement is made using a pair of electrodes transmitting current in the medium and a pair of receiver electrodes measuring the resultant voltage. A dataset ${\textbf {d}}_{\text {meas}}$ of *M* resistances is formed by repeating measurements according to strategies outlined above.
(ii) Once data acquisition is over, the data filtering step is critical to (i) remove measurements from dysfunctional electrodes, (ii) identify and remove outliers in data and (iii) properly assess the error of each measurement, which will be needed during the inversion process.
(iii) The objective of the inversion is to optimize the distribution of resistivity ${\textbf {m}}$ of a discretized medium with *N* cells by reducing the data misfit $\Phi _{\text {d}}({\textbf {m}})$ between the measured dataset ${\textbf {d}}_{\text {meas}}$ and a calculated dataset ${\textbf {d}}_{\text {calc}}$, obtained by simulating measurements for a ER model.
(iv) Once the data misfit $\Phi _{\text {d}}({\textbf {m}})$ between measured and calculated voltages reaches the level of noise of the measured data determined at the pre-processing step, the distribution of resistivity of the model ${\textbf {m}}$ can be considered as a representative image of the true resistivity distribution.

As reported by Tso et al. (2017), the assessment of measurement errors is a critical step to remove outliers, ensure good results for the inversion process and to carry out uncertainty estimation . Several strategies have been developed for error estimations. (i) Contact resistance tests allow the identification of dysfunctional electrodes and the removal of corresponding measurements (Deceuster et al. 2013). (ii) Stacking errors are obtained by calculating the standard deviation of repeated measurements (within a few seconds), which can be done with most commercial resistivity meters (Day-Lewis et al. 2008). (iii) Repeatability errors can be assessed from several measurements typically separated by a few hours. However, repeatability cannot always be assessed in TL-ERT measurements, especially in the case of imaging rapid processes. Finally, (iv) reciprocal errors are calculated by comparing forward measurements with their reciprocal measurements, which are done by exchanging current and potential electrodes (Tso et al. 2017). Although more time is required for completing forward and reciprocal measurements, this option is generally recommended to identify outliers (i.e., configurations that exhibit more than $5~\%$ of difference between forward and reciprocal measurements for instance (Chambers et al. 2014; Tso et al. 2017)) and certain sources of systematic errors. Moreover, this approach provides better estimations of measurement errors, which can be used to define a linear data error model of the form $\varvec{\epsilon } = a + b \cdot {\textbf {d}}_{\text {meas}}$, where *a* and *b* are two constants (Lesparre et al. 2017; Wagner and Wiese 2018) (see. Tso et al. (2017) for a review of *a* and *b* values found in the literature for data error models).

From a theoretical point of view, ${\textbf {d}}_{\text {calc}}$ can be calculated by solving Poisson’s equation (Dey and Morrison 1979) in the medium of heterogeneous ER distribution $\rho$ to compute the voltage distribution *V* caused by a transmitted current *I* at $(x_I, y_I, z_I)$ for each four-electrodes configuration (Rucker 2010):

2 $$\begin{aligned} \frac{\partial }{\partial x} \left( \frac{1}{\rho } \frac{\partial V}{\partial x} \right) + \frac{\partial }{\partial y} \left( \frac{1}{\rho } \frac{\partial V}{\partial y} \right) + \frac{\partial }{\partial z} \left( \frac{1}{\rho } \frac{\partial V}{\partial z} \right) = I ~\delta (x_I) ~\delta (y_I) ~\delta (z_I) \end{aligned}$$

In practice, the forward problem is usually solved with finite element or finite difference methods (Rücker et al. 2006). The forward modeling operation can then be expressed as ${\textbf {d}}_{\text {calc}} = {\textbf {G}}({\textbf {m}})$, given that ${\textbf {m}}$ is the resistivity distribution of the discretized model and ${\textbf {G}}$ is the forward operator (Johnson et al. 2012).

The calculated dataset ${\textbf {d}}_{\text {calc}}$ is then used to compute the data cost-function $\Phi _{\text {d}}$ which will be minimized by the inversion procedure. $\Phi _{\text {d}}({\textbf {m}})$ represents the error between ${\textbf {d}}_{\text {meas}}$ and ${\textbf {d}}_{\text {calc}}$ for the resistivity distribution ${\textbf {m}}$ and is usually expressed by a ***L2*** norm as:

3 $$\begin{aligned} \Phi ({\textbf {m}}) = \left\| {\textbf {W}}_{\text {d}} \cdot ({\textbf {d}}_{\text {calc}} - {\textbf {d}}_{\text {meas}}) \right\| ^2 \end{aligned}$$

where ${\textbf {W}}_{\text {d}}$ is a data weighting matrix calculated from the measurement errors (Lesparre et al. 2017; Singha et al. 2015). It is worth mentioning that other types of regularization terms can be applied such as the ***L1*** norm (also referred to as “robust” inversion), which makes the inversion process less sensitive to data outliers (Auken et al. 2006; Day-Lewis et al. 2006).

Generally, the Gauss-Newton scheme is used to update iteratively the resistivity distribution ${\textbf {m}}$ to reduce $\Phi _{\text {d}}({\textbf {m}})$. The Gauss-Newton procedure can be divided into three steps as follows (Günther et al. 2006) :

(i) The model resistivity distribution is initialized to ${\textbf {m}}_0$, which could be a mean resistivity value or a reference model based on a priori information about the medium (e.g., geological model).
(ii) The resistivity distribution ${\textbf {m}}_0$ is updated into ${\textbf {m}}_1$ according to ${\textbf {m}}_1 = {\textbf {m}}_0 + \Delta {\textbf {m}}$ where the incremental change of resistivity $\Delta {\textbf {m}}$ verifies the normal equation (Günther et al. 2006) :
  4 $$\begin{aligned} {\textbf {J}}^T~{\textbf {W}}_{\text {d}}~{\textbf {J}} \cdot \Delta {\textbf {m}} = {\textbf {J}}^T~{\textbf {W}}_{\text {d}} \cdot ({\textbf {d}}_{\text {calc}} - {\textbf {d}}_{\text {meas}}) \end{aligned}$$
  where ${\textbf {J}}$ is the Jacobian matrix of size *M* x *N* (also called sensitivity matrix) containing the first-order derivative of the calculated data with respect to model parameters.
(iii) The same procedure is repeated from the iteration *k* to the next one by updating resistivity distribution ${\textbf {m}}_{\mathrm {k}}$ according to ${\textbf {m}}_{\mathrm {k}+1} = {\textbf {m}}_{\mathrm {k}} + \Delta {\textbf {m}}$ until (a) the cost function reduction stagnates (Rucker 2010) or (b) the cost function $\Phi ({\textbf {m}}_{\mathrm {k}+1})$ reaches a target value, which typically corresponds to the level of noise of the measured data (Johnson et al. 2012). As a result, many studies use the target value of $\chi ^2=1$ as a stopping criterion for the inversion to prevent data overfitting and underfitting (given $\varvec{\epsilon }$ the error of the *M* measured data ${\textbf {d}}_\text {meas}$) with $\chi ^2$ expressed by: (Johnson et al. 2012)
  5 $$\begin{aligned} \chi ^2 = \frac{1}{M} \sum _{i=1}^{M}\left( \frac{d_{\mathrm {calc,i}} - d_{\mathrm {meas,i}}}{\epsilon _{\text {i}}} \right) ^2 \end{aligned}$$

At the end of the inversion procedure, the resistivity distribution obtained is assumed to be the most representative. However, the inverse problem presented above is non-unique and has an infinite number of solutions. Moreover, the resistivity distribution can be unrealistic, especially when a priori information about the medium is known. This issue is solved by adding to the data cost function $\Phi _{\text {d}}({\textbf {m}})$ a model constraint $\Phi _m({\textbf {m}})$ which penalizes unsuitable ER distributions (Günther et al. 2006). The corresponding cost function can be expressed by the following ***L2*** norm (using Equation 3):

6 $$\begin{aligned} \Phi ({\textbf {m}}) = \left\| {\textbf {W}}_{\text {d}} \cdot ({\textbf {d}}_{\text {calc}} - {\textbf {d}}_{\text {meas}}) \right\| ^2 + \lambda \left\| {\textbf {W}}_{\mathrm {m}} \cdot ({\textbf {m}} - {\textbf {m}}_0) \right\| ^2 \end{aligned}$$

where ${\textbf {m}}_0$ is the reference resistivity distribution, ${\textbf {W}}_{\mathrm {m}}$ is the regularization matrix (e.g., ${\textbf {W}}_{\mathrm {m}} = {\textbf {I}}$ if the model ${\textbf {m}}$ must be close to ${\textbf {m}}_0$) and $\lambda$ is a regularization coefficient (Günther et al. 2006). Similarly to Eq. 3, while the ***L2*** norm corresponds to a smoothness regularization, other norms could be applied such as the ***L1*** norm which favors sharp boundaries to mimic geometrical structures (Auken et al. 2006; Loke et al. 2003) (referred to as “blocky” constraint). As detailed by Johnson et al. (2012), other formulations can be used to ensure maximum, minimum or known resistivities at given locations for example (Johnson et al. 2012). Although these model constraints are necessary to regularize the inversion results and respect a-priori information, several authors noted the risk of (i) overfitting (i.e., fitting data too well subject to model simplifications (Johnson et al. 2012; Wagner and Wiese 2018)) and (i) underfitting (i.e., poor fit between measured and synthetic data due to model constraints that prevent the inversion process from converging toward a suitable resistivity distribution (Tso et al. 2017; Watlet et al. 2018)).

The diagram presented in Fig.  5 can be extended to image subsurface evolution over time if ERT measurements are repeated several times as shown in Fig. 3. A set of *T* datasets $\left( {\textbf {d}}_\text {meas}^{t_1}, ..., {\textbf {d}}_\text {meas}^{t_{\text {T}}} \right)$ are obtained for measurement times $\left( t_1, ..., t_{\text {T}} \right)$ and inverted to recover the resistivity distributions $\left( {\textbf {m}}^{t_1}, ..., {\textbf {m}}^{t_{\text {T}}} \right)$. As discussed by Hayley et al. (2011), several inversion strategies can be used to image resistivity distribution changes over time.

**Independent inversions.** Each dataset can be inverted separately using the methodology presented above; the inverted resistivity distributions would then be compared in absolute values, difference values or relative variations from the starting image. Alternatively, the ratio (or the difference) between consecutive datasets can be considered as input data for the inversion; deviation from 1 (respectively 0) in the inverted images would then be interpreted as an increase or decrease of resistivity (Hayley et al. 2011; LaBrecque and Yang 2001). These approaches are usually referred to as independent inversions (or single snapshot inversions (Karaoulis et al. 2014)) since the result of one time step is independent from the others.

**Time-constrained inversions.** More recent time-lapse inversion strategies impose a similarity between consecutive distributions of resistivity to mimic smooth evolution in time of the medium and discard non-realistic changes of resistivity (Hayley et al. 2011). By analogy with the model constraint presented in Eq. 6, the time constraint $\Phi _{\text {t}}({\textbf {m}}^t)$ can be added to the cost function $\Phi ({\textbf {m}}^t)$ to penalize resistivity distributions ${\textbf {m}}^t$ that differ from the previous one ${\textbf {m}}^{t-1}$. The general expression of $\Phi _{\text {t}}({\textbf {m}}^t)$ is (Loke et al. 2014a; Singha et al. 2015):

7 $$\begin{aligned} \Phi _{\text {t}}({\textbf {m}}^t) = \beta \left\| {\textbf {W}}_{\text {t}} \cdot ({\textbf {m}}^t - {\textbf {m}}^{t-1}) \right\| ^2 \end{aligned}$$

where ${\textbf {W}}_{\text {t}}$ is the temporal regularization matrix (Hayley et al. 2011). Such a time constraint can be applied (i) to invert individual snapshots by using previous inversion results as reference (referred to as “cascade inversion” (Hayley et al. 2011; Singha et al. 2015)) or (ii) to invert simultaneously all datasets which is referred to as “simultaneous” or “4D” inversion (Karaoulis et al. 2014; Kim et al. 2009) (see Kim et al. (2009), Hayley et al. (2011), Karaoulis et al. (2014) and Loke et al. (2014a) for the explicit formulation of 4D inversion).

As discussed by several authors, the resistivity models obtained from time-lapse inversions often need to be corrected to a standard temperature, typically laboratory temperature or mean annual air temperature (e.g., Brunet et al. (2010); Uhlemann et al. (2016b)). This temperature correction is generally expressed by (Hayley et al. 2010) :

8 $$\begin{aligned} \rho ~_{_{\text {T}_{\text {std}}}} = \rho ~_{_{\text {T}}} \cdot \left[ 1 + c \cdot (T-T_{\text {std}}) \right] \end{aligned}$$

where $\rho ~_{_{\text {T}}}$ and $\rho ~_{_{\text {T}_{\text {std}}}}$ are, respectively, the electrical resistivities at temperature *T* and at $T_{\text {std}}$ and *c* is the fractional change in $\rho$ per degree Celsius. Typically, *c* values range between $0.018~^{\circ }\mathrm {C}^{-1}$ and $0.025~^{\circ }\mathrm {C}^{-1}$ (Hayashi 2004; Hayley et al. 2007), which means that electrical resistivity decreases by a factor close to 2 % for a temperature increase of $1~^{\circ }\mathrm {C}$ in the medium (Besson et al. 2008; Chambers et al. 2014). As noted by Uhlemann et al. (2016b), the temperature correction is critical for long-term TL-ERT surveys to correct inversion results for seasonal and diurnal variations of temperature, and avoid misinterpretation of resistivity data.

## Review of TL-ERT Applications Over the Past 30 Years

### TL-ERT Studies Over the Last 30 Years

Several insightful reviews have been dedicated to TL-ERT monitoring over the past 30 years. Samouëlian et al. (2005) established the first review of TL-ERT applications for soil sciences, stressing its strong potential as a non-destructive and large-scale monitoring technique. Slater (2007) described the relationships between electrical and hydrogeological properties of the medium and presented a review of TL-ERT studies to characterize aquifers. Kneisel et al. (2008) reviewed different geophysical methods for permafrost investigations and highlighted the emergence of autonomous TL-ERT as a promising tool for long-term monitoring. Finally, Robinson et al. (2008b) also described the development of TL-ERT applications to bridge the gap between point measurements and large-scale moisture content monitoring from surface techniques (walkover, aerial photogrammetry or remote sensing).

From 2012 to 2015, five well-known reviews have been published to present the developments and perspectives of TL-ERT for subsurface monitoring. Loke et al. (2013) presented a comprehensive overview of measurement system developments, optimized field acquisition methods (2D, 3D and 4D) and data processing techniques. The emergence of hydrogeophysics as a powerful monitoring tool across multiple scales was also discussed by Revil et al. (2012), Binley et al. (2015) and Parsekian et al. (2015). Finally, Singha et al. (2015) published a review describing the geoelectrical monitoring method, from field measurements to data interpretation with various examples of applications .

Since 2014, more than 20 reviews have been published for TL-ERT applications to specific fields such as landslides monitoring (Perrone 2020; Whiteley et al. 2019), ecological applications (e.g., root propagation or water uptake) (Cimpoiaşu et al. 2020; Zhao et al. 2019), geothermal applications (Hermans et al. 2014), salinity issues (e.g., saline intrusion) (Corwin and Scudiero 2019; Costall et al. 2018), infrastructure, buildings or landfill monitoring (Dezert et al. 2019; Romero-Ruiz et al. 2018), groundwater-surface water interactions (Fan et al. 2020; McLachlan et al. 2017) or bioremediation monitoring (Johnson et al. 2015). Falzone et al. (2019) provided a comprehensive review of TL-ERT studies for multiple domains of geosciences involving fluid dynamics monitoring (e.g., hydrogeology, gas flows and contaminant migrations). Wagner and Uhlemann (2021) recently published a review on multi-method geophysical imaging, highlighting promising approaches for the combination of multiple geophysical datasets (including TL-ERT) and numerical models to obtain quantitative estimates of hydrological parameters. Finally, Slater and Binley (2021) recently published a review focusing specifically on advances and perspectives for long-term monitoring of hydrological processes at different scales using TL-ERT.

The present study aims to widen the sphere of these reviews by discussing the strong potential of TL-ERT for mining waste monitoring in the future. A systematic review of TL-ERT applications across various domains from 1991 to 2020 is proposed following the methodology of Aznar-Sánchez et al. (2018) and Zhao et al. (2019). Published studies using TL-ERT (i.e., with at least two ERT snapshots at the same position) have been identified with article searching platforms (Google Scholar, Scopus, Web of Science and Compendex) and the full-texts have been examined to select only the relevant articles. Each relevant article has been classified into the database and its characteristics have been recorded (i.e., year of publication, number of citations, journal, type of application, country, type of TL-ERT measurements, number of electrodes). Finally, the database has been used to classify the different studies and describe the TL-ERT evolution over the past 30 years. A large number of examples of TL-ERT applications in various contexts are used to identify the criteria for success of such TL-ERT surveys. The database is also used to identify the recent developments that could be transferred to future applications of TL-ERT for long-term monitoring of mining wastes. Finally, the present review can serve as a database of TL-ERT studies for various types of application, which can be accessed on the https://adridim.github.io/review2022/0_welcome.html following the example of the catalog of agrogeophysical studies (Blanchy et al. 2020b; Garré et al. 2021; Mary and Blanchy 2021).

**Fig. 6:**
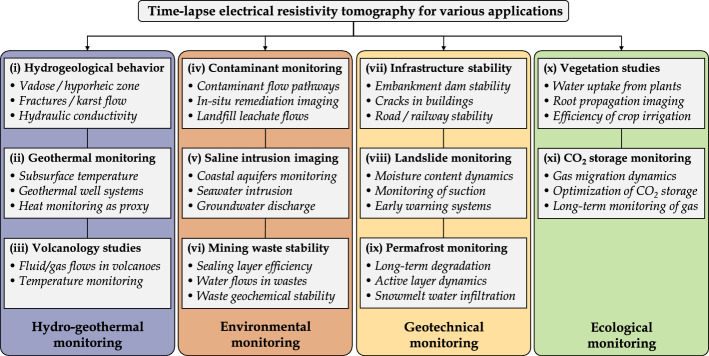
Review of the main applications of TL-ERT for various domains

**Fig. 7:**
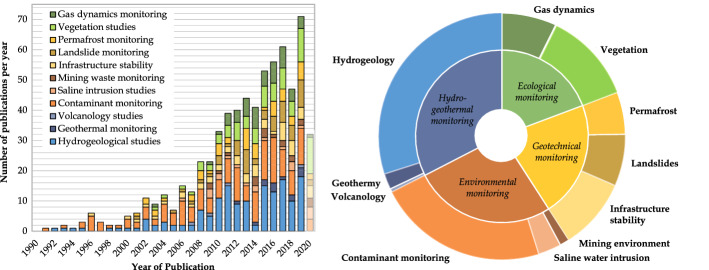
General statistics for each field of applications identified in the database of TL-ERT studies : **a** evolution of the number of published studies per year from 1991 to 2020 for each type of application and **b** distribution of the 651 published studies according to the classification proposed in Fig. 6. Note that the identification of TL-ERT studies has been carried out during Summer 2020, which explains the relatively low number of publications identified for 2020 (shaded)

The diagrams presented in Figs.  6 and 7 review the various fields of applications of the TL-ERT studies identified in the database and present the development of TL-ERT for each of these fields. Each article is classified into four types of monitoring (hydro-geothermal, environmental, geotechnical and ecological) which are themselves divided into three types of applications. Two observations can be made from Fig.  7. On the one hand, the number of publications involving TL-ERT has significantly increased over the past 30 years as reported by the review of Binley et al. (2015) for hydrogeophysics-related applications. Notably, almost 75 % of TL-ERT studies have been published during the last decade, which denotes TL-ERT development over the past years. On the other hand, TL-ERT has been used for an increasing number of fields of applications. While TL-ERT was mostly applied to hydrogeological studies and contaminant monitoring from 1990 to 2010, new types of applications such as ecological applications, permafrost and landslide monitoring have emerged since then. Finally, the pie chart of Fig. 7 presents the distribution of the published studies of the database according to the classification of Fig. 6, highlighting the prominence of hydrogeological and contaminant monitoring applications.

### Description of the Different Fields of Applications for TL-ERT

(i) **Hydrogeological monitoring** corresponds to all applications of TL-ERT used to characterize and monitor the hydrogeological behavior of the subsurface. This field of application accounts for one quarter of the total number of TL-ERT studies in the database and has been a primary application of geoelectrical monitoring from 1990 to 2002 (cf. Figure 7). Since 1990, TL-ERT has been used to image water flow in saturated media (e.g., Busato et al. (2019); Miller et al. (2008)), in the vadose zone (e.g., Parsekian et al. (2015); Petit et al. (2021); Uhlemann et al. (2016a)) or in fractured media (e.g., Carriere et al. (2015); Watlet et al. (2018)). While most studies use the relationship between saturation and electrical resistivity to describe qualitatively the medium behavior (see Eq. 1) (Hübner et al. 2015; Scaini et al. 2017), TL-ERT is sometimes used to characterize quantitatively the medium parameter distribution over large scales such as hydraulic conductivity for instance (Singha et al. 2015; Slater 2007).
(ii) **Geothermal monitoring** is a relatively recent application of TL-ERT, the first occurrence in the literature being studies from Hermans et al. (2012, 2014) on the monitoring of shallow geothermal experiments with TL-ERT . Geothermal monitoring studies aim to recover the spatio-temporal evolution of the subsurface temperature based on its influence on electrical resistivity (see Eq 8.) (Hayley et al. 2007; Ma et al. 2011). This application of TL-ERT is experiencing strong growth since 2015 to describe qualitatively the geothermal behavior of a medium (e.g., Giordano et al. (2016); Robert et al. (2019)) as well as to recover quantitative distribution of temperature in various contexts (e.g., Comina et al. (2019); Lesparre et al. (2019)). Recently, Robert et al. (2019) described the potential of temperature monitoring with TL-ERT as a proxy to study other processes (i.e., use of temperature as a tracer).
(iii) Only a few **volcanology studies** using TL-ERT for volcanoes monitoring have been reported in the literature since 2007. As described by these studies, volcanic activity affects electrical conductivity of the subsurface because of temperature changes, water and gas flows (Di Giuseppe and Troiano 2019; Turner et al. 2011). Although quantitative integration of geophysical, geochemical and geological monitoring datasets is still a challenge, TL-ERT is considered by these authors as a promising monitoring tool to characterize these complex media and processes (Di Giuseppe and Troiano 2019).
(iv) **Contaminant monitoring** refers to all studies using TL-ERT to image the flow of contaminants or tracers which exhibit contrasts of electrical resistivity in comparison with the in-situ pore fluids (see Eq. 1). This type of application accounts for one quarter of the number of TL-ERT studies identified in the database and has been intensively used since the beginning of the 1990s (Bevc and Morrison 1991; Ramirez et al. 1993). While in most studies, the fluids monitored are more conductive than the surrounding pore fluid (e.g., saline fluids (Cassiani et al. 2006; Singha and Gorelick 2005), nitrate plumes (Johnson et al. 2012; Wallin et al. 2013) and leachates from (i) landfills (Audebert et al. 2016; Inauen et al. 2019; Morita et al. 2020), (ii) waste storage ponds (Binley and Daily 2003; Revil et al. 2013) or (iii) olive-oil industry (Simyrdanis et al. 2018)), some contaminants are known to be more resistive (e.g., fresh hydrocarbon plumes (Deng et al. 2017; Trento et al. 2021)). From 1990 to 2010, a vast majority of studies used TL-ERT monitoring either to detect environmental contaminants plumes (Almpanis et al. 2021; Kuras et al. 2016; Power et al. 2015) or to track tracers injected in the subsurface to study its behavior (Koestel et al. 2009). Moreover, TL-ERT has become increasingly popular since the beginning of the 2010s to (i) assess the efficiency of in-situ remediation in contaminated media (Nivorlis et al. 2019; Tildy et al. 2017) or (ii) track leachate recirculation to enhance waste degradation in landfills (Clément et al. 2010; Grellier et al. 2008).
(v) **Saline intrusion monitoring** with TL-ERT aims to characterize and monitor the migration of saline seawater to fresh groundwater or vice versa (Costall et al. 2018). This type of application has been reported since the beginning of the 2010s in Spain (Nguyen et al. 2009; Ogilvy et al. 2009), Italy (De Franco et al. 2009) and USA (Henderson et al. 2010). Most of the studies report electrical resistivity contrasts between groundwater and seawater ranging from one to two orders of magnitude (Costall et al. 2018, 2020). Such high contrasts make TL-ERT a suitable tool (i) to monitor seawater intrusion which could lead to groundwater salinization (Chen et al. 2018; Palacios et al. 2020) and (ii) to characterize submarine groundwater discharge when contaminants flow from land to sea (Henderson et al. 2010; Paepen et al. 2020).
(vi) **Mining waste monitoring** is a relatively uncommon application for TL-ERT since only 20 studies have been identified in the database (less than 3 %). Use of TL-ERT in this context started to be reported in the literature since the beginning of the 2010s (Anterrieu et al. 2010; Maqsoud et al. 2011). Since then, TL-ERT has been used (i) to monitor leachate flows into heap leaching pads in order to maximize mineral recovery (mostly through the work of Rucker (2015) and Rucker et al. (2014)) and (ii) to monitor the geotechnical stability of mining waste storage facilities or mining operations (Mainali et al. 2015; Tresoldi et al. 2020b). Although less common, a few studies have used TL-ERT as a tool (i) to characterize the hydrogeological behavior of mining wastes or storage facilities (Greer et al. 2017; Hester et al. 2019) and (ii) to assess the efficiency of reclamation covers designed to reduce the environmental footprint of mining wastes (Dimech et al. 2019; Maqsoud et al. 2011). As discussed in Sect. 4.1, the scarcity of studies using TL-ERT for mining waste monitoring contrasts with the numerous examples of applications of static ERT imaging in this domain (Martinez-Pagan et al. 2021).
(vii) The potential of TL-ERT for **infrastructure stability** assessment is known since the end of the 1990s with the early study of Johansson et al. (1996) using repeated ERT measurements to monitor the internal erosion and abnormal seepage in dams. Since then, other studies have monitored the increase of electrical resistivity over time in dams or levees as a proxy for voids development due to internal erosion, both at laboratory scale (Masi et al. 2020; Shin et al. 2019) or at field scale (Sjödahl et al. 2008, 2009). Tang et al. (2018) and others applied TL-ERT to evaluate the development of cracks and sinkholes by monitoring the temporal variations of resistivity (Fabregat et al. 2017; Samouëlian et al. 2004). As discussed in the review of Smethurst et al. (2017), TL-ERT is also becoming increasingly popular to monitor abnormal water accumulation, infiltration or seepage in structures since these processes usually correspond to a decrease in ER. Such resistivity anomalies can then be used as a proxy for deterioration of geotechnical stability (i) in earth embankments and levees (Arosio et al. 2017; Tresoldi et al. 2019) (ii) in railway embankments and road structures (Chambers et al. 2014; Rasul et al. 2018) or (iii) in buildings (De Donno et al. 2017; Voss et al. 2019).
(viii) **Landslide monitoring** programs have recognized TL-ERT as an efficient, economical and complementary tool since the beginning of the 2000s (Lebourg et al. 2005; Supper et al. 2008). Recently, this type of application has experienced strong growth. Indeed, more than half of the studies identified in the database have been published during the past five years. Nowadays, TL-ERT is considered as one of the most popular geophysical techniques for long-term landslide monitoring and several recent reviews are available in the literature (Perrone 2020; Whiteley et al. 2019). From a qualitative point of view, TL-ERT has been used as early as 2006 to characterize the internal structure of landslides (e.g., Crawford and Bryson (2018); Huntley et al. (2019)) and to assess their hydrogeological behavior (e.g., Chen et al. (2018); Peng et al. (2019)). In addition, many studies have used TL-ERT in a quantitative way since 2015 to recover (i) landslide displacements (Boyle et al. 2018; Uhlemann et al. 2015; Wilkinson et al. 2016), (ii) moisture content distribution (Gance et al. 2016; Gunn et al. 2015; Uhlemann et al. 2017) and (iii) suction stress distribution (Crawford et al. 2019; Hen-Jones et al. 2017). As discussed by Whiteley et al. (2019), such applications have a great potential in the future for the understanding of processes that trigger landslide activation (e.g., Merritt et al. (2018); Uhlemann et al. (2017)), making TL-ERT a promising tool for landslide long-term monitoring and early warning (e.g., Holmes et al. (2020); Supper et al. (2014)).
(ix) **Permafrost monitoring** with TL-ERT has been reported in the literature since the early 2000s, mostly with the studies of Hauck (2001) and Hauck et al. (2003). The authors identified TL-ERT as a promising and robust method to monitor permafrost dynamics since unfrozen water, ice and air have strong electrical resistivity contrasts that can be well resolved by ERT (Hauck 2001). Since then, TL-ERT has been used (i) to image the internal structure of permafrost (Dafflon et al. 2017; Fortier et al. 2008), (ii) to track snowmelt infiltration (French and Binley 2004; Thayer et al. 2018), (iii) to monitor unstable permafrost rock walls (Keuschnig et al. 2017; Krautblatter et al. 2010), (iv) to study the dynamics of freeze-thaw processes in the active layer (Farzamian et al. 2019; Murton et al. 2016) and (v) to monitor long-term permafrost degradation (Mewes et al. 2017; Mollaret et al. 2019). Today, TL-ERT is recognized as a mature, efficient and robust tool for long-term permafrost studies. For instance, some sites in the Alps have been monitored with autonomous remote TL-ERT for more than 20 years (Etzelmüller et al. 2020; Mollaret et al. 2019). Notably, recent developments have focused on quantitative monitoring of ice, water, air and rock content from TL-ERT and seismic monitoring (Mollaret et al. 2020; Wagner et al. 2019) as well as improvement of semi-empirical relationships (Herring et al. 2019; Toran et al. 2013).
(x) **Vegetation studies** use TL-ERT to monitor the soil-plant-atmosphere interactions from repeated resistivity measurements (Garré et al. 2021). This type of application is known since the beginning of the 2000s with the studies of Michot et al. (2003) and Al Hagrey (2006); al Hagrey et al. (2004). Since then, TL-ERT has become a popular tool in this context. More than 60 % of the 80 studies referenced in the database have been published during the past five years. As described by Garré et al. (2021), many studies have used TL-ERT to monitor the water dynamics around and in the plants at different scales using petrophysical relationships described above (Eq. 1) (Michot et al. 2003). Such applications range from (i) the characterization of root-water uptake (Dick et al. 2018; Garré et al. 2013; Mary et al. 2020), (ii) the circulation of water within trunks and stems (Al Hagrey 2006; Luo et al. 2019) to (iii) the assessment of water budget for the soil/plant/atmosphere system (Brillante et al. 2015; Cassiani et al. 2016; Vanella et al. 2019). Moreover, TL-ERT has been used for agriculture purposes (i) to monitor water availability for crops (Beff et al. 2013; Brillante et al. 2016a), (ii) to assess the plant response to drought or unconventional irrigation (Carrière et al. 2020; De Carlo et al. 2019) or (iii) to monitor the performance of crop irrigation (Hardie et al. 2018; Vanella et al. 2021). Finally, several studies have used TL-ERT to monitor root propagation, both at the laboratory scale (e.g., Corona-Lopez et al. (2019); Weigand and Kemna (2019)) and in the field (e.g., Mary et al. (2016); Whalley et al. (2017)).
(xi) **Gas dynamics monitoring** with TL-ERT has been reported since the early 2000s with the study of Ramirez et al. (1993) where the infinite electrical resistivity of gas was used to track carbon dioxide flow inside experimental tanks. Since then, this approach has been popular for various applications as 50 TL-ERT studies have been published since 2010. TL-ERT has been used (i) to monitor induced carbon dioxide flooding during oil extraction (Ramirez et al. 1993; White et al. 2011), (ii) to track gas flows in landfills (Rosqvist et al. 2011), and (iii) to study carbon dioxide or methane migration in the subsurface (e.g., Klazinga et al. (2019); Kremer et al. (2018)). As reported by the review of Bergmann et al. (2016), TL-ERT has also been used as a long-term surveillance tool for carbon dioxide sequestration, for example at the Ketzin $\text {CO}_2$ storage pilot site in Germany (Bergmann et al. 2012; Schmidt-Hattenberger et al. 2016). TL-ERT is promising in this regard since abnormal resistivity increases can be interpreted as $\text {CO}_2$ leaks (Dafflon et al. 2013; Yang et al. 2019).

## Emergence, Challenges and Perspectives of TL-ERT for Mining Wastes

### Review of Promising Applications of TL-ERT for Mining Wastes

Although TL-ERT has been recognized as a valuable tool to provide additional information about subsurface processes in many domains, there are few examples of applications for mining wastes monitoring, either for short or long periods of time. This observation is all the more striking considering (i) the need for efficient mining waste monitoring techniques and (ii) the numerous applications of single-time ERT in mining wastes, as reported by Martinez-Pagan et al. (2021), the first review of ERT applications in this context. Therefore, the review below aims to identify promising avenues for TL-ERT monitoring of mining wastes.

**Fig. 8:**
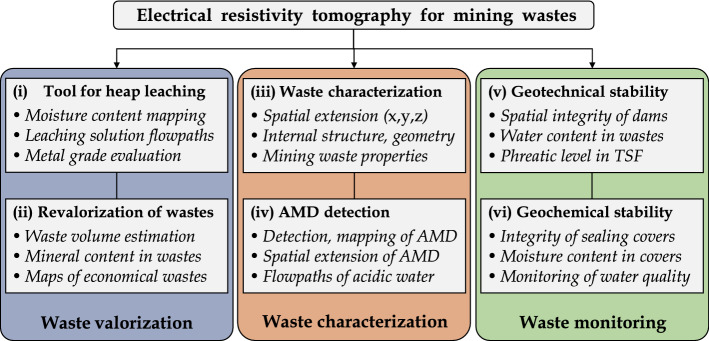
Review of the main applications of ERT for mining wastes imaging and monitoring

The methodology presented in Sect. 3.1 has been followed to build a database of published studies using ERT for mining wastes since 1990, with most studies published over the last decade. Figure 8 presents the different types of applications of ERT in mining wastes identified from the database. Each study has been classified into six types of applications, themselves classified into three broader domains: waste valorization, waste characterization and waste monitoring. A brief overview of each type of application is proposed below (i) to present the objective of ERT measurements in mining wastes, (ii) to discuss about the advantages and limitations of ERT in each context, and finally (iii) to identify promising applications for TL-ERT monitoring on mining wastes.

(i) ERT has been used as a **support tool for mineral extraction** in heap leaching pads (HLP) since the beginning of the 2010s, mostly through the work of Rucker et al. (Rucker et al. 2009a, 2017) (c.f. Table 1). As described by Maghsoudy et al. (2019), heap leaching is a common mineral extraction process. Leaching solutions are injected into ore piles to mobilize metals which are then collected through drainage pipe networks (Rucker 2010). ERT has been applied to track leaching solutions flowpath since these solutions are usually highly conductive (Maghsoudy et al. 2019; Rucker et al. 2009a). As a result, ERT has been used to improve heap leaching efficiency by (i) imaging heterogeneous water distribution in HLPs (Rucker 2010), (ii) estimating remaining fractions of metals (Rucker et al. 2009c, 2017) and (iii) evidencing HLP heterogeneity effects on macropore flows, leachate solution accumulation or dry areas (Maghsoudy et al. 2019). These studies provide valuable insights for (i) advanced ERT instrumentation strategies in mining wastes (large-scale imaging (Cubbage et al. 2016; Rucker et al. 2017), borehole-surface layouts (Rucker 2014)) as well as for (ii) data-processing and interpretation developments for mining waste monitoring (real-time monitoring (Rucker et al. 2014), quantitative interpretation (Rucker 2010), and complex 3D visualization (Rucker et al. 2017)). Such strategies can be transferable to future studies on WRPs to provide large-scale and non-destructive information that could help (i) to assess their hydrogeological behavior (e.g., Dimech et al. (2019)), (ii) to track meteoritic water flows or tracers within WRP (e.g., Hester et al. (2019)) or (iii) to delineate and monitor acidic water flows within mining wastes (e.g., Bortnikova et al. (2018)).
(ii) Applications of ERT for **revalorization of mining wastes** have been reported since 2016 with the study from Günther and Martin (2016). The authors used ERT and spectral induced polarization to map mineralized areas in a mining slag heap for potential future mineral reuse (Günther and Martin 2016). Since then, ERT has been applied to reconstruct the spatial distribution of mining wastes over large scales and estimate the remaining mineralization, both for WRPs (Martin et al. 2020; Qi et al. 2018) and TSFs (Martínez-Segura et al. 2020; Saladich et al. 2016) (c.f. Table 2). In most cases, a threshold in electrical resistivity has been defined to delineate the mineralized wastes, which are generally more conductive (Martin et al. 2020; Saladich et al. 2016). Moreover, ERT has been combined with aerial photogrammetry or LIDAR surface topography to recover the volume of mineralized mining wastes (Markovaara-Koivisto et al. 2018; Martín-Crespo et al. 2018). These results are of great interest for the development of circular economy in mining. Indeed, the knowledge about spatial distribution and mineralization is identified as a limiting factor for mining waste reuse as discussed by Kinnunen and Kaksonen (2019). It is worth noting that the methodology followed by these studies could be applied in the future (i) to provide complementary data for systematic geochemical sampling of mining wastes (e.g., Elghali et al. (2019); Gabarrón et al. (2020)) or (ii) to identify areas that could be prone to contaminated drainage generation due to high mineralization (e.g., Epov et al. (2017); Power et al. (2018)).
(iii) **TSF and WRP characterization** using ERT has been increasingly popular since the early studies of (i) Martínez-Pagán et al. (2009) for TSF characterization and (ii) Poisson et al. (2009) and Anterrieu et al. (2010) for WRP internal structure investigations (c.f. Table 3). Since then, ERT has been used (i) to determine the spatial extension of old/abandoned WRPs (Martin et al. 2020) and TSFs (Martín-Crespo et al. 2020, 2018) and (ii) to image the internal structure and heterogeneity of WRPs and TSFs (Martinez-Pagan et al. 2021; Mollehuara-Canales et al. 2021). In particular for WRPs, the coarse waste rocks have been reported to be more resistive than the compacted layers of fine particles (Anterrieu et al. 2010; Poisson et al. 2009), which has led to a better understanding of WRP hydrogeochemical behavior (e.g., Martin et al. (2017); Raymond et al. (2021)). Similarly, the tailings are usually more conductive than the surrounding media, which allows mapping properly the bedrock interface and the dams used to contain tailings (e.g., Booterbaugh et al. (2015); Gabarrón et al. (2020)). Notably, Gabarrón et al. (2020) used ERT to differentiate coarse and fine tailings in a TSF from their contrast of resistivity. Many authors have also used ERT along with geochemical analysis to map potentially reactive wastes (e.g., Dimech et al. (2017); Power et al. (2018) in WRPs and (Martínez et al. 2012, 2016) in TSFs) and even quantitatively predict (i) total dissolved solids (TDS) (Epov et al. 2017; Rucker et al. 2009a), (ii) pH (Yurkevich et al. 2017), or (iii) the concentration of various contaminants in tailings (e.g., Canales et al. (2020); Vásconez-Maza et al. (2019)). Finally, a few studies have used ERT to characterize the hydrogeological behavior of mining wastes (i) by assessing relationships between electrical resistivity and the hydrogeological properties of tailings (Banerjee et al. 2011; Canales et al. 2020) and (ii) by tracking water flows in WRPs under natural or artificial precipitation events (Dimech et al. 2019; Greer et al. 2017). Although less common, these types of applications have great potential for mining waste characterization since TL-ERT provides large-scale data that allows taking into account WRP and TSF heterogeneous composition in a non-destructive manner (Hester et al. 2019; Poisson et al. 2009) and recover information where no other hydrogeological data is available, such as in the core of WRPs (Dimech et al. 2019; Greer et al. 2017).
(iv) Studies of **acid mine drainage (AMD)** with ERT have been reported in the literature since the 1990s, both for AMD detection (Benson and Addams 1998; Ebraheem et al. 1990) and AMD monitoring (Buselli and Lu 2001; King and McNeill 1994) (c.f. Table 4). AMD is generally associated with high concentrations of metallic ions in the pore water (Blowes et al. 2014; Cravotta III 2008), which increases the electrical conductivity of pore water (up to several orders of magnitude (Monterroso and Macías 1998)). As a result, AMD is a suitable target for ERT imaging and TL-ERT monitoring (Buselli and Lu 2001; Johnston et al. 2017), which enables the detection and delineation of areas within TSFs or WRPs where contaminated drainage is generated (e.g., Bortnikova et al. (2013); Shokri et al. (2022); Tycholiz et al. (2016)). Such information has been used (i) to extend spatially geochemical sampling (Martínez-Pagán et al. 2009; Pierwoła et al. 2020) and (ii) to help design future reclamation covers which would reduce the environmental impacts of mining wastes (Hudson et al. 2018; Martínez-Pagán et al. 2009). Similarly, ERT has been used (i) to track AMD migration within TSFs (Bortnikova et al. 2018; Hudson et al. 2018) and WRPs (Casagrande et al. 2020; Shokri et al. 2016a) and (ii) to identify the mechanisms of contaminants transport from the storage facilities to the surrounding medium (e.g., preferential flow of contaminated water (Bethune et al. 2015; Casagrande et al. 2018; do Nascimento et al. 2022), fractures flow inside the bedrock (Benyassine et al. 2017; Olenchenko et al. 2016), eolian transport (Lachhab et al. 2020) or leaks from sealing layers (Cortada et al. 2017; Rey et al. 2020)). As illustrated by the recent study from Puttiwongrak et al. (2019), semi-permanent TL-ERT installations have a strong potential for the long-term monitoring of AMD. Permanent electrode arrays could be installed near or within the mining wastes to track electrical resistivity changes over large scales, hence increasing the capacity of traditional monitoring techniques. Electrical resistivity could then be used as a proxy indicating possible AMD generation or migration from the wastes, as it has already been done in the past for other types of contaminants (e.g., Denham et al. (2020); Heenan et al. (2015)).
(v) Application of ERT for **geotechnical stability** assessment of TSFs has been reported since 2005 with the early study from Sjödahl et al. (2005) in TSF dams (c.f. Table 5). Since then, ERT has been used in TSFs (i) to detect anomalous seepage and internal erosion within the dams (Coulibaly et al. 2017; Li et al. 2015; Mainali et al. 2015; Paria et al. 2020; Sjödahl et al. 2005) and (ii) to image water table elevation in the TSFs, which can help to manage the risk of water overtopping (Booterbaugh et al. 2015; Mainali 2006; Mainali et al. 2015). Such applications have a great potential for geotechnical stability monitoring of TSFs since seepage, dam erosion and overtopping have been the cause of almost 60 % of TSF failures worldwide since 1910, as detailed by the comprehensive review of Lyu et al. (2019b). Moreover, several studies have combined ERT and geotechnical modeling to study the stability of WRPs (Li et al. 2015) and TSFs (Coulibaly et al. 2017; Paria et al. 2020) as it has already been done for landslide monitoring (Lehmann et al. 2013; Zieher et al. 2017) or levee monitoring (Dezert et al. 2019; Weller et al. 2014). As discussed by Sjödahl et al. (2005), TL-ERT monitoring has great potential in this context since repeated ERT images allow tracking changes in resistivity across large scales, which can reduce ambiguities in ERT data interpretation. Following the recent developments of TL-ERT for real-time monitoring of landslides, permanent arrays could be installed within TSFs to monitor remotely their geotechnical stability as part of early warning systems (Kłosowski et al. 2018; Whiteley et al. 2019). As discussed by Tresoldi et al. (2020a), such applications of TL-ERT are expected to become increasingly popular given raising awareness toward the environmental and human risks that TSFs and WRPs can represent.
(vi) ERT has been used for **geochemical stability** assessment of mining wastes since the beginning of the 2000s (Bergström 1998; Binley and Daily 2003) (c.f. Table 6). As discussed by Bussière and Guittonny (2020a), most reclamation approaches aim to control oxygen or water flows toward the mining wastes with engineered covers installed on TSFs or WRPs since AMD generation is mostly controlled by the oxidation of the sulfide contained in wastes (Mbonimpa et al. 2020; Plante et al. 2020). ERT has been mostly used in this context (i) to assess the efficiency of mining reclamation by detecting AMD generation on reclaimed TSFs (Power et al. 2018; Rucker et al. 2009a), (ii) to detect leaks from sealing layers (such as geotextiles) used to encapsulate reactive tailings (Binley and Daily 2003; Villain et al. 2015), and lastly (iii) to provide insights about how reclamation designs could be improved (Acosta et al. 2014; Rey et al. 2020; Sylvain et al. 2019). Although less common, several studies also used TL-ERT to monitor the efficiency of multilayer covers made of granular materials which offer better durability than geotextiles (e.g., store and release covers (Bussière and Wilson 2020), covers with capillary barrier effects (Demers and Pabst 2020a) or flow control layers (Demers and Pabst 2020b)). For instance, Maqsoud et al. (2011) used TL-ERT to ensure that a retention layer made of fine materials remained near saturation over time, which would allow reducing oxygen migration from the atmosphere toward the tailing storage facility, hence decreasing the risk of AMD generation. More recently, TL-ERT has been used to track water flows in an experimental WRP to assess the performance of a flow control layer designed to divert water from the reactive core of the WRP (Dimech et al. 2019; Martin et al. 2019). Semi-permanent TL-ERT monitoring systems could be used along with traditional monitoring techniques to provide early warnings if reclamation does not meet the initial design objectives or if its performance decreases over time (e.g., Bussière et al. (2020); Dimech et al. (2021)).

**Table 1:** Review of studies using ERT to monitor metal extraction in heap leaching pads (HLP)

References	Country	TSF / WRP	ERT setup	Remarks
Rucker et al. (2009c)	United States Nevada	WRP HLP	pseudo-3D ERT 160m x 420m	3D imaging of HLP Conversion of $\rho$ into gold with drill-core sampling
Rucker et al. (2009b)	United States Nevada	WRP HLP	2D ERT lines (x2) 150m to 240m STAR array (x2)	Monitoring of moisture content changes over time Leachate solution pathways
Rucker (2010)	United States Nevada	WRP HLP	pseudo-3D ERT 160m x 420m	3D estimation of moisture content in HLP from ERT data with cokriging
Rucker et al. (2014)	United States Colorado	WRP HLP	3D STAR array Boreholes (x6)	Real-time monitoring of reagent pathways in HLP
Cubbage et al. (2016)	United States Arizona	WRP HLP	2D ERT lines (x50) Boreholes (x20)	Operational use of TL-ERT to optimize metal extraction
Rucker et al. (2017)	United States Arizona	WRP HLP	2D ERT Lines (x4) 400m to 850m Boreholes (x24)	3D TL-monitoring of reagent Estimation of total and extracted copper in HLP
Maghsoudy et al. (2019)	Iran Kerman	WRP HLP	2D ERT Lines (x5) 200m to 400m	Identification of macropore flow, dry areas and water accumulation in the HLP

**Table 2:** Review of studies using ERT to estimate the volume of mining wastes for valorization

References	Country / State	TSF / WRP	ERT setup	Remarks
Florsch et al. (2012)	France Occitanie	WRP *Slag heap*	pseudo-3D ERT 25m x 18m	3D imaging of a slag heap for estimation of archaeological metal production (130 tons)
Günther and Martin (2016)	Germany Lower-Saxony	WRP *Slag heap*	2D ERT line 42m	Use of ERT and SIP to map mineralized areas for reuse
Saladich et al. (2016)	Spain Catalonia	TSF	2D ERT lines (x10) 50m to 100m	Threshold in $\rho$ to discriminate bedrock, cover and tailings
Markovaara-Koivisto et al. (2018)	Finland Western Finland	TSF	pseudo-3D ERT 200m x 300m	Threshold in $\rho$ to identify high-grade copper wastes LIDAR data for volume
Nikonow et al. (2019)	Chile Santiago	TSF	2D ERT lines (x13) 120m to 1300m	3D distribution of fine/coarse grained tailings Correlation with copper
Martin et al. (2020)	Germany Lower-Saxony	WRP	2D ERT lines (x27) 25m to 99m	Sampling data to estimate threshold in $\rho$ for wastes with economic potential
Martínez-Segura et al. (2020)	Spain Murcia	TSF	2D ERT lines (x16) 85m to 215m	Thresholds in $\rho$ to estimate depth of wastes and volume estimation with LIDAR data

**Table 3:** Review of some studies using ERT to characterize mining wastes and storage facilities

References	Country	TSF / WRP	ERT setup	Remarks
Poisson et al. (2009)	Canada Quebec	WRP	pseudo-3D ERT 36m x 30m	Identification of fine-grained and coarse-grained layers Validation with two trenches
Anterrieu et al. (2010)	Canada Quebec	WRP	2D ERT lines (x12) 26m to 160m	Large-scale internal structure of WRP from ERT inversions History of WRP construction
Martinez et al. (2014)	Spain Andalucia	TSF	2D ERT lines (x2) 400m to 480m	Large-scale internal structure of TSF and assessment of environmental risk
Booterbaugh et al. (2015)	Canada Alberta	TSF	2D ERT lines (x3) 600m to 800m	Comparison of TL-ERT with direct measurements of water content and EC Identification of water table
Martínez et al. (2016)	Spain Andalucia	TSF	2D ERT line 290m	Combined ERT and hydrogeochemical internal characterization of TSF
Epov et al. (2017)	Russia Kemerovo	TSF	2D ERT lines (x3) 235m	Mapping of reactive tailings Relationship between $\rho$ - $\theta$, sulfide content and TDS
Greer et al. (2017)	United States Virginia	WRP *Valley fill*	2D TL-ERT lines 90m to 315m	TL-monitoring of water infiltration (artificial event) Hydrogeological behavior
Yurkevich et al. (2017)	Russia Kemerovo	TSF	2D ERT lines (x6) 14m to 33m	Delineation of oxidized tailings with ERT $\rho$ - pH relationship
Martín-Crespo et al. (2018)	Spain Murcia	TSF	2D ERT lines (x6) 165m to 260m	TSF history and tailing volumes estimated from ERT and aerial photogrammetry
Power et al. (2018)	Canada Nova-Scotia	WRP	2D ERT lines (x7) 140m to 365m pseudo-3D ERT 140m x 80m	Internal composition of WRP Validation with logging Delineation of AMD and future sources of AMD
Hester et al. (2019)	United States Virginia	WRP *Valley fill*	2D TL-ERT lines 45m to 422m	TL-monitoring of water infiltration (artificial event) Hydrogeological behavior
Martín-Crespo et al. (2019)	Spain	TSF	Multiple 2D ERT 45m to 500m	Review of ERT imaging for 7 abandoned TSF in Spain
Vásconez-Maza et al. (2019)	Spain Murcia	WRP/TSF	2D ERT lines (x4) 90m to 215m	Prediction of Chromium content from $\rho$ values with $\rho$ - Cr in-situ relationship
Gabarrón et al. (2020)	Spain Murcia	TSF	2D ERT lines (x7) 10m to 180m	Systematic sampling and high-resolution ERT to assess various relationships
Canales et al. (2020)	Australia South Australia	TSF	2D ERT line 410m	Sampling of tailings to assess petrophysical relationship with fluid EC and saturation
Vásconez-Maza et al. (2020)	Spain Murcia	WRP/TSF	2D ERT lines (x8) 90m to 215m	Classification of wastes with PCA (Principal Component Analysis) of ERT results

**Table 4:** Review of the most recent studies using ERT to characterize AMD flows from mining wastes

References	Country	TSF / WRP	ERT setup	Remarks
Pierwoła (2015)	Poland Małopolska	TSF	2D ERT lines (x2) 27m to 90m	Delimitation of Zn-Pb tailings prone to AMD Gradation in sulfide content
Shafaei et al. (2016)	Iran Semnan	WRP	2D ERT lines (x4) 90m to 170m	TL-ERT to track AMD plume Identification of AMD source
Shokri et al. (2016b)	Iran Semnan	WRP	pseudo-3D ERT 65m x 75m	Modeling of $O_2$ diffusion to validate correlation between low $\rho$ and $FeS_2$ oxidation
Tycholiz et al. (2016)	Canada Manitoba	TSF	VES (x2)	VES and electromagnetic used to map pH and $Cu^{2+}$ Comparison with systematic sampling of tailings (2D)
Benyassine et al. (2017)	Morocco Drâa-Tafilalet	TSF	2D ERT lines (x5) 300m to 1200m	Imaging of potential AMD flowpaths in abandoned TSF Identification of fractures
Johnston et al. (2017)	United States Colorado	TSF	2D ERT line 500m	ERT mapping of potential diffuse sources of AMD Comparison with local geochemical and water EC
Casagrande et al. (2018)	Brazil Minas Gerais	WRP	pseudo-3D ERT 600m x 900m	Large-scale 3D mapping of AMD affected water Design of future reclamation
Bortnikova et al. (2018)	Russia Kemerovo	TSF	pseudo-3D ERT 350m x 500m	AMD flowpaths imaged with large-scale 3D-ERT Tailing volume calculation
Hudson et al. (2018)	United Kingdom Wales	TSF	2D ERT lines (x8) 100m to 120m	Mapping of contaminated streams, calculation of heavy metal efflux, identification of remediation strategies
Targa et al. (2019)	Brazil Minas Gerais	WRP	2D ERT lines (x4) 400m	Identification of flowpaths for AMD through fractures Mapping of sulfides in WRP
Casagrande et al. (2020)	Brazil Minas Gerais	WRP	pseudo-3D ERT 600m x 900m	Large-scale 3D mapping of AMD affected water Design of future reclamation
Lachhab et al. (2020)	Morocco Drâa-Tafilalet	TSF	2D ERT lines (x3) 630m to 790m	ERT combined with seismic to identify AMD pathways from TSF through fractures
Martín-Crespo et al. (2020)	Spain Murcia	WRP/TSF	2D ERT lines (x2) 120m	Combination of aerial photogrammetry and ERT to map AMD occurrence
Moreira et al. (2020)	Brazil Minas Gerais	WRP	pseudo-3D ERT 300m x 350m	Mapping of AMD affected water and preferential flow through fractured bedrock
Pierwoła et al. (2020)	Poland Małopolska	TSF	2D ERT lines (x2) 50m to 500m	Delineation of contaminated areas from AMD products and eolian transport

**Table 5:** Review of studies using ERT to evaluate the geotechnical stability of WRP and TSF

References	Country / State	TSF / WRP	ERT setup	Remarks
Sjödahl et al. (2005)	Sweden Närke	TSF	2D ERT lines (x4) 150m to 825m	Imaging of TSF internal structure (tailings, dam core) Low $\rho$ values for the area with known stability issues
Li et al. (2015)	China Jilin	TSF/WRP	2D ERT lines (x4) 50m to 240m	Identification of loose waste rocks and saturated tailings from $\rho$ for stability analysis
Coulibaly et al. (2017)	Canada Quebec	TSF	2D ERT lines (x2) 260m to 340m	Heterogeneity observed in the dam, relationship with geotechnical stability
Paria et al. (2020)	Peru	TSF	2D ERT lines (x4) 330m to 560m	Mapping of TSF geometry and underlying material
Tresoldi et al. (2020a)	Chile Santiago	TSF	2D ERT lines (x3) 150m	TL autonomous monitoring Field trial to test a system prior to TSF installation

**Table 6:** Review of studies using ERT to evaluate mining wastes reclamation efficiency

References	Country / State	TSF / WRP	ERT setup	Remarks
Rucker et al. (2009a)	United States Montana	WRP/TSF Open-pit	pseudo-3D ERT 600m x 500m	3D delineation of AMD in a reclaimed TSF validation with sampling
Martín-Crespo et al. (2010)	Spain Andalucia	TSF	2D ERT lines (x6) 50m to 190m	Identification of acidic water flows in the TSF ($\rho <1~\Omega$m) Good performance of sealing
Maqsoud et al. (2011)	Canada Quebec	TSF	2D ERT line 30m	Accumulation of water in the retention layer of a cover with capillary barrier effects
Acosta et al. (2014)	Spain Murcia	TSF	2D ERT lines (x9) 175m to 355m	Evidence of erosion of the cover layers (wind or water) Good quality of bedrock
Villain et al. (2015)	Sweden Norrbotten	WRP Open-pit	2D ERT lines (x4) 200m to 280m	Existence of seepage within a dry cover, risk of erosion
Cortada et al. (2017)	Spain Andalucia	TSF	2D ERT lines (x4) 190m to 315m	High-moisture zones interpreted as faults in the insulation of the tailings
Dimech et al. (2018)	Canada Quebec	WRP	3D TL-ERT 60m x 10m x 7m	Moisture content monitoring in a reclamation cover Laboratory calibration
Dimech et al. (2019)	Canada Quebec	WRP	3D TL-ERT 60m x 10m x 7m	Monitoring of water flows in an experimental WRP Validation with point sensors
Rey et al. (2020)	Spain Andalucia	TSF	2D ERT lines (x2) 180m to 390m	Detection of acidic leaks from the reclaimed TSF

**Fig. 9:**
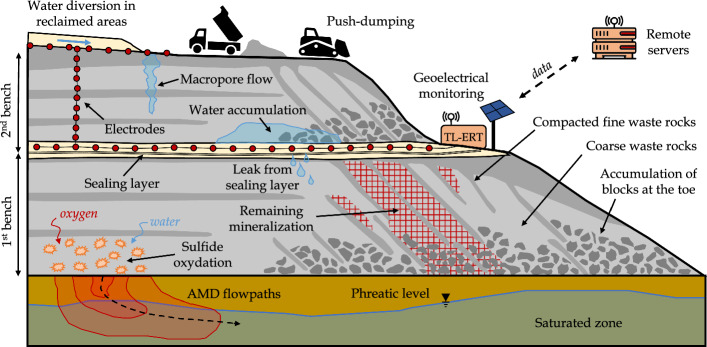
Review of the key parameters that can be imaged or monitored in WRPs with TL-ERT as identified from the database of ERT studies in mining wastes (adapted from Aubertin et al. (2005))

**Fig. 10:**
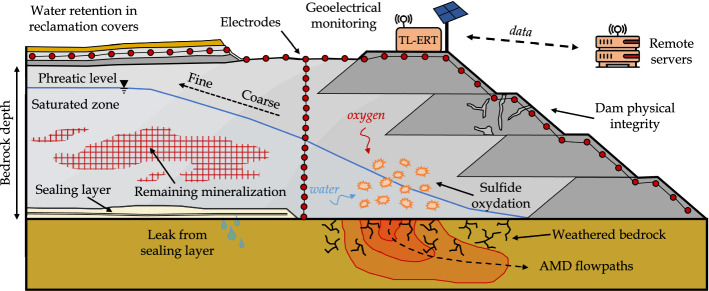
Review of the key parameters that can be imaged or monitored in TSFs with TL-ERT as identified from the database of ERT studies in mining wastes (adapted from Aubertin et al. (2016))

In summary, Figs.  9 and 10 review graphically the different parameters that have been imaged with ERT in WRPs and TSFs, respectively. These figures highlight the types of application for which TL-ERT is the most promising for both short-term and long-term monitoring of mining wastes as discussed above. The diagrams of the internal structures of WRPs and TSFs on Figs.9 and 10 are inspired by the work of Aubertin et al. (2005) and Aubertin et al. (2016).

### Challenges that Need to be Addressed to Improve TL-ERT Monitoring in Mining Wastes

Although TL-ERT has been identified as a promising non-destructive imaging approach for monitoring of various dynamic processes in WRPs and TSFs, several challenges are likely to emerge from a wider adoption in mining wastes. The following section discusses three categories of challenges that have been identified for future TL-ERT applications on WRPs and TSFs. (i) Mining sites are generally complex and often remote sites, in constant evolution with harsh field conditions. (ii) Both high spatial and temporal resolutions are needed while maintaining reliable and consistent monitoring over large scales for long periods of time. (iii) Many physical parameters can change simultaneously in mining wastes, although high accuracy of measurements is generally needed for mining waste monitoring. For a broader discussion on advantages and limitations of TL-ERT, the reader is referred to the comprehensive studies of Samouëlian et al. (2005), Binley et al. (2015), Parsekian et al. (2015), Soupios and Kokinou (2016), McLachlan et al. (2017) and Whiteley et al. (2019).

(i) **Complex sites and harsh field conditions**
  Firstly, mining sites present generally harsh field conditions for long-term monitoring programs since some mines are in remote locations, with few road access and limited power supply, especially after the end of mining operations. In addition, when mining operations are over, monitoring instruments could be damaged by wildlife, vandalized, or stolen, which would undermine the success of long-term monitoring and could represent high maintenance costs. Even active mines can represent challenging conditions because of security limitations, circulation of heavy machinery and rapid evolution of the site that may affect measurements (e.g., change of electrodes, cables and resistivity meters location or update of topography models). In addition, the instrumentation of WRPs and TSFs is highly challenging in itself. Indeed, WRPs are built by high benches (typically between 10 m and 30 m in height) with strong slopes, contain large blocks (typically over one meter in size), are highly heterogeneous and can exhibit electrical resistivity over 10 k$\Omega$m (Dimech et al. 2017; Vriens et al. 2020a). As reported by several authors, such internal structure makes it extremely challenging to install any sensor or electrode within the core of WRPs, and may cause long-term data quality issues due to poor electrode grounding or cable deterioration (Deceuster et al. 2013; Greer et al. 2017). Although TSFs are generally more homogeneous structures with better electrode contact conditions (fine and conductive material, typically less than 100 $\Omega$m (Martinez-Pagan et al. 2021)), the installation of ERT monitoring systems might be challenging as well if the tailings are too wet to allow operators to walk on them (e.g., MEND (2000)). Finally, the long-term durability of electrodes and cables in mining wastes has still not been studied, but it is likely that such pieces of equipment could suffer from corrosion issues in media with low pH and high EC (Palacios et al. 2020; Peter-Borie et al. 2011).
(ii) **High spatio-temporal resolution for long time periods at large scales**
  Secondly, the spatio-temporal characteristics needed for monitoring programs on mining wastes are also especially challenging since high spatial and temporal resolution are generally required over large distances and during long periods of times (Bussière et al. 2020; MEND 2004). From the temporal perspective, suitable resolution (i.e., sampling rate) depends on the process studied as discussed in Sect. 2.2. For instance, high resolution is needed to properly recover water infiltration into mining wastes (e.g., one snapshot every 30 minutes, typically during a few days (Dimech et al. 2019; Hester et al. 2019)), while a lower resolution might be sufficient to recover long-term desaturation of a moisture-retaining layer or an abnormal accumulation of water within a TSF (e.g., one snapshot every day, typically during several years (Dimech et al. 2021; Tresoldi et al. 2020a)). Although there is no consensus about how long TSFs and WRPs monitoring programs should last, the geotechnical and geochemical stability of these mining waste storage facilities must be ensured for hundreds of years (MEND 2004). Since TL-ERT is a relatively new monitoring technique, the longest monitoring period recorded in the literature is approximately 20 years (Etzelmüller et al. 2020; Mollaret et al. 2019). As a result, it is challenging to predict the lifetime of a monitoring system (electrode, cables, battery, resistivity meter), and future work might be necessary to improve the robustness of TL-ERT systems in the context of mining wastes to prevent electrode loss, long-term drifts and noise issues (Peter-Borie et al. 2011; Watlet et al. 2018). From the spatial perspective, monitoring large areas such as TSFs and WRPs generally requires to find a compromise between spatial extension, electrode number and spatial resolution (see Sect. 2.2). Moreover, since most processes summarized in Figures 9 and 10 occur in the shallow subsurface (typically in the first ten meters), small electrode spacing (such as 1 m or 2 m) might be needed to monitor them accurately. As a result, for a single TL-ERT profile of 1 km across a TSF or a WRP, between 500 and 1000 electrodes might be needed, which is challenging both in terms of data acquisition system capacity, monitoring costs, time needed for each snapshot, power supply needed for measurements and volume of data (Falzone et al. 2019; Parsekian et al. 2015). Given that in Canada alone, almost 200 mining operations have TSFs or WRPs that exceed 1 km$^2$, upscaling TL-ERT monitoring systems while ensuring a decent spatial resolution at shallow depths is expected to be one of the main challenges for a broader application of this technique to mining wastes. In the meantime, new strategies must be developed to define optimized location for the TL-ERT monitoring systems, which would allow monitoring the stability of TSFs and WRPs where it is crucial.
(iii) **Simultaneous evolution of many physical parameters affecting electrical resistivity**
  Lastly, as reported by the review of Vriens et al. (2020a), many physical processes can occur simultaneously at different scales in mining waste storage facilities (e.g., various geochemical reactions, water and air advection, dispersion and diffusion). As a result, several physical parameters that affect electrical resistivity can vary simultaneously and have confounding effects (e.g., temperature, moisture content, ice content, pore water EC, pH, porosity), which could make the conversion of electrical resistivity imaged by TL-ERT into a physical parameter useful for geotechnical or geochemical stability monitoring challenging (Dimech et al. 2019; Parsekian et al. 2015). On the opposite, some hydrogeological or geotechnical properties of the mining wastes might not have a direct influence on electrical resistivity (such as the interstitial pressure or the hydraulic conductivity, for instance). Moreover, the relative scarcity of petrophysical models developed for mining wastes reported by Canales et al. (2020) increases the uncertainty and variability of petrophysical approaches discussed by Tso et al. (2019) and others. Such limitations could have consequences on the accuracy of quantitative interpretations derived from TL-ERT, especially since they are combined with the well-known limitations of ERT data inversion (e.g., non-uniqueness of inversion (Samouëlian et al. 2005), inversion artifacts (Carey et al. 2017; Greer et al. 2017), non-uniform sensitivity distribution and spatial resolution (Clément et al. 2010; Rucker 2014)). This issue is all the more challenging given the need for high accuracy for monitoring techniques in mining wastes, since slight changes of moisture content (for instance) could correspond to significant deterioration of WRPs and TSFs geotechnical and geochemical stability (Bussière et al. 2020; Demers and Pabst 2020a; MEND 2004).

### Perspectives for Future Applications of TL-ERT on Mining Waste Monitoring

**Fig. 11:**
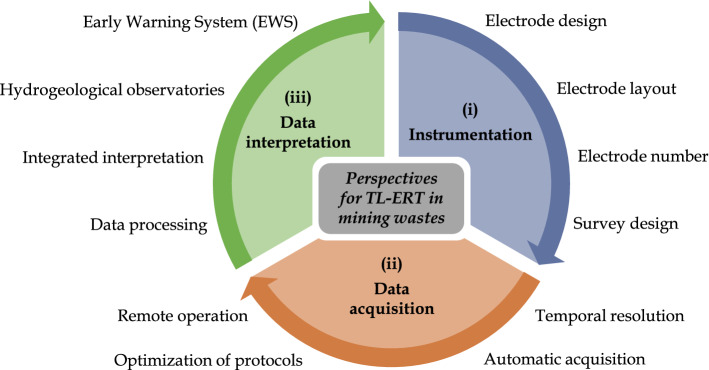
Recent developments and perspectives for geoelectrical monitoring of mining wastes

The lessons learned from the numerous TL-ERT studies can provide valuable insights to address, at least in part, the challenges mentioned above for long-term monitoring of mining waste stability. For instance, several studies using TL-ERT for long-term monitoring of permafrost noted the complexity of ensuring good data quality in frozen soils and in remote areas (e.g., Tomaškovičová et al. (2016)). The strategies that have been developed to address such issues in permafrost might be suitable as well for waste rocks, for instance. More generally, the “tremendous progress made by the geoelectrical method over the past 25 years” described by Loke et al. (2013) and by other reviews since then (e.g., Binley et al. (2015); Singha et al. (2015)) can help to identify promising avenues for the application of TL-ERT in WRPs and TSFs. The following section summarizes these technological advances in regard to the TL-ERT studies that carried out long-term monitoring with semi-permanent installations. In particular, several perspectives are proposed for instrumentation, data acquisition and data interpretation as illustrated by Fig. 11.

**Fig. 12:**
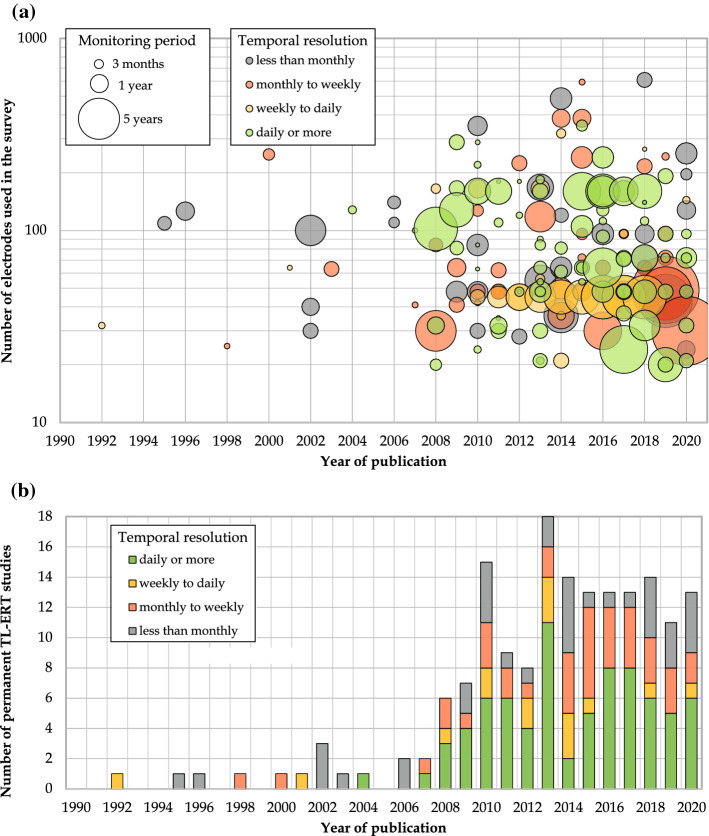
**a** Evolution of the number of electrodes, the monitoring period and the temporal resolution for the 173 semi-permanent TL-ERT studies identified in the database. For each study, the size of the circle is proportional to the monitoring period while the color of the circle corresponds to the temporal resolution. **b** Histogram of semi-permanent TL-ERT studies according to the temporal resolution

**Fig. 13:**
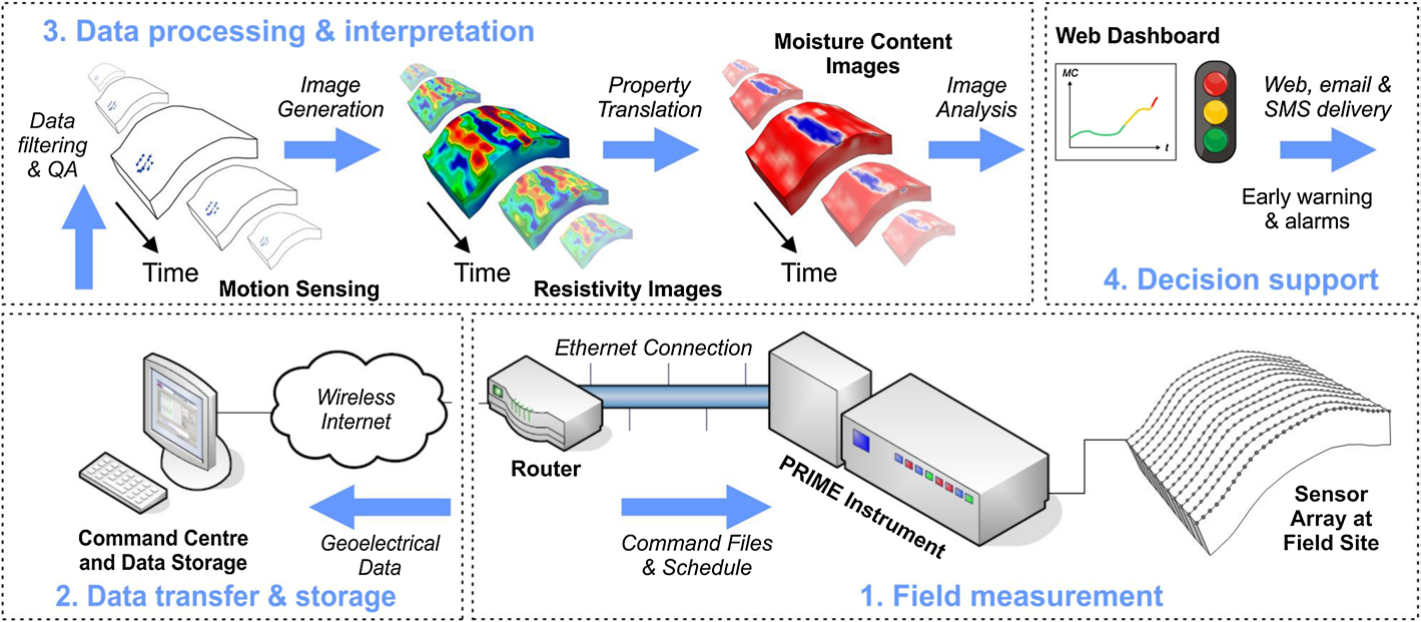
Flowchart of permanent TL-ERT monitoring system describing autonomous data acquisition, remote data transfer, automated processing and interpretation for long-term monitoring. Figure inspired from Holmes et al. (2020) presenting the workflow of the PRIME system applied to landslide monitoring in British Columbia (Canada)

- **Electrode design** (i.e., shape, size and material) is recognized by many authors as a possible source of erroneous measurements if the contact resistance between the electrodes and the medium is too high (e.g., in the presence of rocks, dry medium, macropores or ice) (Dick et al. 2018; Oldenborger and LeBlanc 2018). This issue could be particularly challenging for long-term ERT monitoring in mining wastes because the deterioration of the electrode-ground contact over time can lead to misinterpretation of the data and increase the cumulative error of the measurement (Deceuster et al. 2013). Recently, Tomaškovičová et al. (2016) carried out a long-term TL-ERT study in arctic conditions to compare several designs of electrodes. Their results evidenced (i) that plate electrodes ensure better grounding than vertical rods and (ii) that mesh electrodes further improve coupling with the medium thanks to a larger effective surface area. As a result, buried plate electrodes (or disks) are becoming increasingly common for long-term studies to increase electrical grounding and protect electrodes from animals, human or vegetation disturbance (Arosio et al. 2017; Tresoldi et al. 2019). Large mesh plates and robust cables should be favored for future long-term monitoring of WRPs since the contact resistance in waste rocks is expected to be high and to minimize the risk of cable damage due to large rocks (e.g., Dimech et al. (2019); Tresoldi et al. (2020a)). Moreover, the electrodes should be buried during the construction of TSFs and WRPs or inside small trenches to further improve the grounding following the example of Tresoldi et al. for long-term mining wastes monitoring (Tresoldi et al. 2020b, a). Notably, the vast majority of TL-ERT studies referenced in the database have used stainless-steel electrodes to reduce long-term corrosion (LaBrecque and Daily 2008). However, few studies have focused on the long-term corrosion effects on electrodes in acidic conditions that could be found in some TSFs or WRPs. We suggest that future studies could focus on identifying strategies to improve the durability of electrodes and cables in mining wastes, following the methodology developed by Tomaškovičová et al. (2016).
- **Electrode layout** in the field is also an important aspect of TL-ERT, since it determines the spatial extension, the depth of investigation and the spatial resolution (Loke et al. 2013). While most semi-permanent TL-ERT studies have used standard 2D lines of electrodes (buried or not) (Brunet et al. 2010; De Franco et al. 2009), it is worth noting that about a third of the semi-permanent monitoring studies have used more complex electrode layouts to improve spatial resolution where needed (Loke et al. 2013). On the one hand, several TL-ERT surveys have used (i) 3D grids of electrodes (Chambers et al. 2011; Uhlemann et al. 2016a), (ii) borehole electrodes in 2D or 3D (Carrigan et al. 2013; Kiessling et al. 2010) or (iii) combination of surface and borehole electrodes (Clément et al. 2010; Kiflu 2016). Future studies could take advantage of the high modularity of TL-ERT layouts in order to maximize the sensitivity of geoelectrical monitoring where needed within WRPs and TSFs (Rucker 2014; Rucker et al. 2009c). On the other hand, half of long-term TL-ERT surveys of the database have favored permanent electrodes buried in the medium rather than standard surface arrays to (i) improve electrode grounding (Busato et al. 2019; Tresoldi et al. 2019), (ii) reduce errors associated with electrode mislocation (Peter-Borie et al. 2011; Wilkinson et al. 2015a), (iii) protect electrodes and cables from animal or human deterioration (Auken et al. 2014; Perrone et al. 2014), (iv) minimize disturbance of the medium during measurements (Jayawickreme et al. 2010) and (v) reduce time and labor costs needed to install electrodes for each snapshot (French et al. 2002; Peter-Borie et al. 2011). The latter could be done for long-term monitoring of mining wastes given the risk of human and animal deterioration in remote locations, especially after the end of mining operations. Finally, most authors agree on the importance of electrode surveying to accurately include electrode positions and topography within inversion models (e.g., Uhlemann et al. (2016a); Wilkinson et al. (2015a)).
- The **number of electrodes** used for ERT surveys has increased significantly over the last 30 years, which in turn has improved spatial resolution and/or spatial extension (Loke et al. 2013). Following the example of Whiteley et al. (2019), Fig. 12 presents for each semi-permanent TL-ERT study (i) the year of publication (x-axis), (ii) the number of electrodes used (y-axis), (iii) the duration of the monitoring period (size of the circle) and (iv) the temporal resolution (color of the circle). This plot illustrates that long-term monitoring studies using more than 100 electrodes for daily measurements have become common practice since the end of the 2000s (e.g., Nguyen et al. (2009); Ogilvy et al. (2009)). Since 2010, several TL-ERT studies with more than 250 electrodes are reported in the literature. Such studies benefit from increased electrode capacity to carry out (i) high spatial resolution monitoring surveys (with electrode spacing as low as 50 cm (Dahlin et al. 2014; Palacios et al. 2020)) or (ii) large scale monitoring surveys covering up to $1.5~\text {km}$ in length (Auken et al. 2014; Ulusoy et al. 2015). Such improvements are of great interest for future applications on large-scale monitoring of mining waste storage facilities since they allow to increase the monitored area while maintaining a decent spatial resolution. In particular, TL-ERT surveys with improved spatial resolution and spatial extension represent a strong potential for a better characterization of material heterogeneity within mining wastes (Dick et al. 2018; Slater and Binley 2021). For instance, the spatio-temporal dynamics of electrical resistivity across WRPs and TSFs could be used to identify distinct regions that exhibit different behaviors, following the unsupervised classification approach of Whiteley et al. (2021), Watlet et al. (2018), McLachlan et al. (2020) and Delforge et al. (2021).
- **Survey design** has been recognized by some authors as a critical step for the successful application of long-term TL-ERT (Robinson et al. 2019; Slater and Binley 2021). For instance, Slater and Binley (2021) stressed the need to know “what to measure and when” for long-term resistivity monitoring systems to ensure that the spatio-temporal resolution is suitable for the dynamic process monitored. In practice, the latter can be done with feasibility studies that evaluate the potential effectiveness of TL-ERT surveys prior to actual field measurements. Following the example of Robinson et al. (2019), modeling tools could be used to simulate the spatio-temporal dynamics of a specific subsurface process (e.g., migration of contaminants). Synthetic TL-ERT datasets can then be compared to identify the electrode spacing, electrode location, electrode layout, measurement protocol or temporal sampling that are the most appropriate to image properly the dynamic process of interest (cf Sect. 2.2). Such feasibility studies have been carried out either (i) numerically (e.g., Klazinga et al. (2019); Mewes et al. (2017)), (ii) with laboratory experiments in columns or in tanks (e.g., Hojat et al. (2020); Kremer et al. (2018)) or even (iii) with simplified field surveys (e.g., Tresoldi et al. (2020a)). We suggest that similar approaches should be developed to improve the design of future TL-ERT surveys in mining wastes, given the complexity of these sites, both in terms of geometry and material properties. Moreover, prior hydro-geothermal modeling and reactive transport modeling in mining wastes could (i) help to identify the physical parameters that are likely to change in the wastes over time and (ii) estimate the corresponding spatio-temporal changes in electrical resistivity. These modeling results could allow identifying TL-ERT survey designs that are the most likely to provide satisfying monitoring results in TSFs or WRPs. As discussed by Henderson et al. (2010), such preliminary studies could help reduce the risk of installing poorly designed ERT monitoring systems on the field, which would represent missed opportunities and significant costs. Last but not least, the location of TL-ERT surveys within TSFs and WRPs must be determined appropriately, since these structures usually cover several hundreds of hectares and could not be entirely monitored. It might be relevant to develop methodologies to identify critical areas of TSFs and WRPs where long-term ERT monitoring would be most needed depending on the slope, vegetation cover, material property, heterogeneity and water table elevation for instance (Bussière et al. 2020).
- In recent years, the **temporal resolution** of semi-permanent TL-ERT monitoring has improved, both for (i) the duration of TL-ERT monitoring studies (denoted as “monitoring period” on Fig. 12) as well as (ii) for the interval between each ERT snapshot (i.e., temporal resolution). As illustrated in Figure 12, the monitoring period of semi-permanent TL-ERT studies has significantly increased since the first applications in the 1990s (size of the circles). Indeed, 20 studies with continuous TL-ERT data covering 5 years or more have been published since 2010 (e.g., Schmidt-Hattenberger et al. (2017); Uhlemann et al. (2016a)). For instance, Mollaret et al. (2019) and Etzelmüller et al. (2020) reported the longest acquisition period (20 years) for permafrost monitoring. In the meantime, the temporal resolution has also increased (color of the circles in Fig. 12), which has been allowed by low-power resistivity meter developments that allow carrying out several measurements per day while being powered by off-grip power systems (Holmes et al. 2020). As shown by Figure 12, more than half of the semi-permanent studies published in the last decade have acquired at least one ERT snapshot per day (e.g., Chambers et al. (2008); Nguyen et al. (2009)). While in most cases, a trade-off needs to be found between the spatial extension/resolution (i.e., electrode number), the temporal resolution and the monitoring period, some studies have demonstrated that TL-ERT monitoring systems can handle hundreds of electrodes while acquiring ERT snapshots daily during several years (e.g., Johnson et al. (2015); Kuras et al. (2016)). Such improvements are particularly promising for long-term monitoring of large-scale TSFs and WRPs, given the need for both high spatio-temporal resolution and large monitored areas discussed above. For instance, horizontal profiles containing several hundred of electrodes could be used to monitor WRPs a TSFs for decades with a temporal resolution greater than one image per day.
- **Automatic resistivity meters** have been greatly improved since the start of their commercialization in the 1990s (Binley and Slater 2020; Loke et al. 2013). As discussed by many authors, resistivity meter recent developments include an increased number of electrodes and data-storage capacity, the development of multi-channel measurement, an improved data quality with reduced power consumption and a better robustness for harsh field conditions (Binley and Slater 2020; McLachlan et al. 2017). The resistivity meters used in the 173 semi-permanent surveys presented in Fig. 12 can be classified into two categories. On the one hand, several resistivity meters, well-known for single-time acquisition, have been adapted to carry out long-term monitoring (e.g., Syscal (Arosio et al. 2017; Palis et al. 2017) or ABEM instruments (Bièvre et al. 2018; Caterina et al. 2017)). On the other hand, several resistivity meters have been designed for long-term, autonomous and remote monitoring since the end of the 2000s (e.g., ALERT (Kuras et al. 2009; Ogilvy et al. 2009)), GEOMON-4D (Supper et al. 2008, 2014)), PRIME (Holmes et al. 2020; McLachlan et al. 2020) and GRETA systems (Arosio et al. 2017; Tresoldi et al. 2019)). Such resistivity meters are particularly well suited for long-term monitoring in remote areas such as mining sites since they are self-powered (with solar panels or wind turbines), they need less power for measurements and they can transfer data remotely (Slater and Binley 2021). Finally, Binley and Slater (2020) noted the growing interest in low-cost and open-source resistivity meters (Sherrod et al. 2012), such as the OhmPi instrument (Clement et al. 2020).
- **Optimized data acquisition protocols** for ERT surveys have been studied since the beginning of the 2000s with the work of Stummer et al. (2004), Furman et al. (2007) and Wilkinson et al. (2006). By definition, optimized protocols are measurement protocols which maximize the spatial resolution of each ERT survey with the minimal number of four-electrode measurements, thus reducing the time needed to perform each ERT snapshot (e.g., Palacios et al. (2020); Qiang et al. (2022); Wilkinson et al. (2006)). Since then, several studies have been published to improve optimized protocols design by (i) taking into account multi-channel capacity (Wilkinson et al. 2012), (ii) minimizing electrode polarization effects (Wilkinson et al. 2012) and (iii) managing complex electrode layouts such as borehole electrodes (Loke et al. 2014b), 3D ERT surveys (Loke et al. 2014c), combined surface and buried arrays (Loke et al. 2015a) and large numbers of electrodes (Loke et al. 2015b). Optimized protocols are particularly promising for TL-ERT monitoring of dynamic processes (such as in mining wastes) since they allow to increase both spatial and temporal resolution, especially for surveys with large numbers of electrodes and/or unconventional layouts (Binley and Slater 2020; Wilkinson et al. 2015b). Finally, recent studies have demonstrated the potential of simultaneous optimization of measurement protocols and electrode location, which ensures maximal TL-ERT spatio-temporal resolution while reducing the number of electrodes needed, and the instrumentation costs (Uhlemann et al. 2018; Wagner et al. 2015). Moreover, Wilkinson et al. (2015b) have developed a methodology to further improve spatio-temporal resolution by updating the measurement protocols over time in order to maximize ERT resolution where temporal changes are observed . Such approaches could be useful in the context of TSFs and WRPs monitoring to design optimized electrode layouts and help improve temporal and spatial resolution where and when it is the most needed (e.g., after heavy rains or in unstable areas).
- **Remote operation** of resistivity meters has become increasingly popular since the beginning of the 2010s (Binley and Slater 2020; Versteeg and Johnson 2013). Autonomous resistivity meters can be connected to electrode arrays and left on-site during several years (Holmes et al. 2020). They are usually installed in protective housing and powered by solar panels or wind turbines, which allows carrying out autonomous measurements throughout the year in remote areas (e.g., Holmes et al. (2020); Merritt et al. (2018)). A wireless Internet connection can be used to remotely upload command files, schedule measurements and download data files from the resistivity meters to remote servers (Holmes et al. 2020). It is worth noting that external sensors such as rain gauge or moisture sensors can be connected to resistivity meters to detect specific meteorological conditions and trigger higher temporal resolution TL-ERT acquisition (Binley and Slater 2020). Figure 13 presents one of these autonomous resistivity meters; the PRIME system (see Holmes et al. (2020) for details). These developments have a great potential for long-term monitoring of mining wastes since they reduce the frequency of installation, maintenance and data acquisition field campaigns, which can be expensive, time-consuming or even not possible at all for extreme conditions or remote mining sites (French et al. 2002; Uhlemann et al. 2021). Moreover, this type of autonomous monitoring system allows for near real-time data transfer, processing and can send alerts (Web, email or SMS) if predefined thresholds of moisture content (or other parameters) are exceeded, which could be highly valuable to detect any deterioration of the geochemical or geotechnical stability of mining wastes.
- **Data processing** tools and frameworks for TL-ERT have experienced strong growth over the last years, which in turn increased the reliability of geoelectrical monitoring (Binley and Slater 2020). On the one hand, several modeling and inversion algorithms have been developed to allow TL-ERT data processing with large 2D or 3D models, using complex boundaries and advanced spatio-temporal constraints (e.g., R2/R3t (Binley and Slater 2020), pyGIMLi (Günther et al. 2006; Rücker et al. 2017), ResIPy (Blanchy et al. 2020a; Boyd et al. 2019) and E4D (Johnson et al. 2010, 2012)). These features are necessary to increase the accuracy of inversion results, especially in complex media such as in TSFs or WRPs. It is worth mentioning that most of these recent software are open-source, free for academic use, and are usually based on well-known numerical computing platforms such as MATLAB®  or python (e.g., Eidors (Adler and Lionheart 2006; De Donno and Cardarelli 2017), pyGIMLi or SimPEG (Cockett et al. 2015; Heagy et al. 2017)). Moreover, some of these recent modeling and inversion tools have been adapted to link TL-ERT to (i) hydrogeological, thermal and reactive transport modeling (e.g., PFLOTRAN-E4D (Johnson et al. 2017; Tso et al. 2020)), or (ii) other geophysical methods (e.g., pyGIMLi and SimPEG). In particular, TL-ERT datasets could be combined to modeling tools widely used for mining wastes such as MIN3P-HCP (e.g., (Raymond et al. 2020; Vriens et al. 2020b)), which would allow comparing the spatio-temporal dynamics of electrical resistivity with the predicted thermal, hydrogeological and chemical behavior of TSFs and WRPs. Such approaches have a strong potential for mining waste monitoring since TL-ERT datasets would extend spatially the area covered by conventional sensors and help to validate the predicted behavior of TSFs and WRPs across larger scales. On the other hand, automated processing workflows have been developed, which help to reduce the time needed for traditional data filtering, data processing and inversion (Versteeg and Johnson 2013; Watlet et al. 2018). These autonomous data processing techniques are all the more promising given the increasing size of long-term LT-ERT datasets. Finally, the review from Khan and Ling (2019) noted that alternative approaches such as machine learning techniques are emerging for TL-ERT data processing (e.g., artificial neural networks (Kłosowski et al. 2018; Rymarczyk et al. 2019) or random forest algorithms (Brillante et al. 2016b)). Moreover, data assimilation techniques such as ensemble Kalman filters are especially promising for the integration of TL-ERT into conventional monitoring programs for various applications (including mining waste monitoring) and to estimate model uncertainties (e.g., Bouzaglou et al. (2018); Camporese et al. (2015); Kang et al. (2018); Tso et al. (2020); Vereecken et al. (2008)). In the context of mining wastes, data assimilation techniques would then allow combining TL-ERT datasets and conventional hydrogeological sensors with multi-physical modeling tools to predict more accurately the behavior of these complex media.
- **Integrated interpretations** of TL-ERT datasets are increasingly popular for the monitoring of key physical parameters such as water, gas or ice content (Michot et al. 2003; Rucker 2010), temperature (Hermans et al. 2018; Herring et al. 2019), suction (Crawford et al. 2019; Lehmann et al. 2013) or contaminant concentration (Deng et al. 2017; Doetsch et al. 2012) in the subsurface. As discussed in Sect. 2.1, the empirical petrophysical approach introduced by Archie in the 1940s (Archie et al. 1942) for oil reservoirs remains widely used by TL-ERT studies and recent work has been done to refine and improve our physical comprehension of these relationships (e.g., Cai et al. (2017); Glover (2015)). Quantitative approaches are of great interest for mining waste monitoring in particular given that all the physical parameters mentioned above could play a role in mining waste geotechnical and geochemical stability (Bussière et al. 2020; MEND 2004). As discussed in the review of Friedman (2005), it is generally recommended to determine site-specific petrophysical relationships for each TL-ERT survey to improve the precision of key physical parameter estimation. These calibrations are usually done with simultaneous and co-located measurements of the physical property to be recovered and electrical resistivity in the laboratory (i) in core samples (< 10 L) (Corona-Lopez et al. 2019; Hen-Jones et al. 2017), (ii) in columns (< 100 L) (Dimech et al. 2018; Priegnitz et al. 2013) and (iii) with tank experiments (> 100 L) (Bechtold et al. 2012; Lyu et al. 2019a; Roodposhti et al. 2019). We suggest that future studies could focus on improving petrophysical models for mining wastes, following the recent examples of Canales et al. (2020), Wayal et al. (2021) and others. In particular, the development of predictive petrophysical relationships from basic geotechnical properties (e.g., grain size distribution, porosity) and geochemical properties (e.g., mineralogy) seems promising given that such information is widely available in the literature and for most mining sites (Aubertin et al. 2003; Mbonimpa et al. 2003). Moreover, the development of standardized and replicable procedures to calibrate petrophysical relationships in the laboratory seems highly promising since it would allow comparing different datasets and different mining wastes from various sites (e.g., Chen et al. (2018); Ling and Zhang (2017)). Finally, direct in-situ characterization with hydrogeological sensors and TL-ERT has also been increasingly popular and we suggest that similar approaches could be used more frequently under field conditions in mining wastes (e.g., Crawford and Bryson (2018); Watlet et al. (2018)). Although the calibration of site-specific relationships improves the accuracy of moisture content estimations from TL-ERT, most authors warn against solely relying on TL-ERT to predict moisture content (Dimech et al. 2019; Tso et al. 2019). For instance, a recent study from Tso et al. (2019) highlighted the importance of validating TL-ERT results with other monitoring techniques (such as moisture content sensors) or with hydrogeological modeling. In this regard, coupling between hydrogeological, thermal and geophysical modeling has been used to (i) explicitly integrate the dependency of electrical resistivity on temperature and moisture content and (ii) take into account the hydrogeo-thermal behavior of the materials (e.g., Kuhl et al. (2018); Wagner and Wiese (2018)). In practice, this type of approach is promising in mining wastes since it would allow the quantification of the uncertainty of petrophysical relationships on moisture content values for instance (e.g., Tso et al. (2019)), and would help to carry out sensitivity analysis on TL-ERT results (Brunet et al. 2010). Finally, the recent review from Wagner and Uhlemann (2021) discussed the development of multi-method geophysical imaging which allows the uncertainty of inversion to be reduced. For instance, TL-ERT could be combined with seismic imaging in TSFs or WRPs to improve the estimation of water content, ice and porosity, following the recent examples of Wagner et al. (2019) and Mollaret et al. (2020) for permafrost monitoring. Among other geophysical methods that could be combined with TL-ERT monitoring of mining wastes, passive seismic methods are particularly promising since they provide complementary information with a low power consumption for long-term monitoring (e.g., Olivier et al. (2017); Planès et al. (2016); Whiteley et al. (2019)). Distributed acoustic sensing cables could be installed along with TL-ERT electrode cables across large-scale profiles in TSFs and WRPs, which would allow the simultaneous monitoring of electrical resistivity and seismic velocities in mining wastes (e.g., Bakulin et al. (2020); Mollehuara-Canales et al. (2021); Verdon et al. (2020)). Finally, it is worth mentioning that other electrical methods such as self-polarization, induced polarization or spectral-induced polarization are promising for mining wastes monitoring since they are affected by different physical parameters of interest (e.g., Mainali et al. (2015); Saneiyan et al. (2019)).
- The integration of TL-ERT into **hydrogeological observatories** has been identified by Slater and Binley (2021) and other authors as a key to success for the future development of long-term electrical monitoring studies (Parsekian et al. 2015). Indeed, hydrogeological observatories are well-known sites that are generally heavily instrumented using multiple monitoring techniques across different spatio-temporal scales (Jensen and Refsgaard 2018; Robinson et al. 2008a). For instance, such observatories have played a critical role in the recent development of long-term TL-ERT monitoring of landslides processes (e.g., Hollin Hill site in the UK (Boyd et al. 2021; Uhlemann et al. 2017)), for CO$_2$ storage monitoring (e.g., Ketzin site in Germany (Bergmann et al. 2016; Schmidt-Hattenberger et al. 2017)), contaminant monitoring (e.g., Hanford site in the USA (Johnson et al. 2015; Robinson et al. 2019)) and for permafrost studies (e.g., Murtel site in the Alps (Mollaret et al. 2019; Supper et al. 2014)). We suggest that a similar approach could be followed for the monitoring of geotechnical and geochemical stability of mining wastes with experimental WRPs and TSFs observatories in the future. For instance, critical sections of TSFs and WRPs could be instrumented with a dense network of conventional monitoring instruments, TL-ERT profiles and other geophysical methods (e.g., Dimech et al. (2019); Martin et al. (2019)). These multi-physical datasets could be used to (i) validate and improve TL-ERT monitoring results under field conditions, (ii) to test new integrated data processing approaches, (iii) to assess the advantages of TL-ERT as a complementary monitoring technique over large scales and lastly (iv) to support long-term research programs and train future hydrogeophysicists in the context of mining waste geochemical and geotechnical monitoring. Moreover, such large-scale field tests would be “proofs of concept” to demonstrate the feasibility and the value of TL-ERT for mining waste monitoring. As discussed by Parsekian et al. (2015), such effort could be helpful to address what can be identified as a primary challenge; “the resistance to adoption of geophysical measurements” from operators that “have relied on traditional measurements of the subsurface,” who can be “hesitant to adopt new technologies that may be viewed as untested.”
- **Early Warning Systems** (EWS) are “monitoring devices designed to avoid or to mitigate the impact posed by a threat” (Medina-Cetina and Nadim 2008). The potential of semi-permanent TL-ERT monitoring to provide additional information for existing EWS has been identified since the end of the 2000s with the studies from Kuras et al. (2006) and Ogilvy et al. (2009) in the context of saline intrusion monitoring. Since then TL-ERT has been part of EWS for many geo-hazards surveillance programs, although none has been reported for mining wastes. The main examples of applications are (i) landslide prediction and surveillance (Budler 2017; Smethurst et al. 2017; Supper et al. 2014), (ii) dam and levee geotechnical stability monitoring (Arosio et al. 2017; Tresoldi et al. 2020b), (iii) railway embankment stability monitoring (Chambers et al. 2014; Gunn et al. 2018), (iv) unstable permafrost rock wall surveillance (Keuschnig et al. 2017; Weber et al. 2019), (v) monitoring of saline water intrusion into groundwater aquifers (Chen et al. 2018; Ogilvy et al. 2009), (vi) surveillance of flash flooding from both natural causes or human activities (El-Saadawy et al. 2020; Liu et al. 2017) and (vii) monitoring of contaminant leaks from industrial and mining sites (Denham et al. 2020; Puttiwongrak et al. 2019). Such an approach is promising for mining wastes since TL-ERT monitoring could be used for the early detection of any condition associated with the deterioration of geotechnical or geochemical stability of WRPs and TSFs (e.g., sudden change of water table elevation, abnormal seepage in TSF dams, abnormal increase or decrease in moisture content or sudden increase of pore water electrical conductivity). In this regard, some on-going projects are currently testing long-term TL-ERT monitoring for the assessment of mining waste geotechnical and geochemical stability at pilot scale (e.g., Kłosowski et al. (2018); Tresoldi et al. (2020a)). We suggest that future monitoring studies could define critical threshold values (either for moisture content or electrical resistivity). Alerts could then be transferred to the operators if these values are exceeded, which would help to prevent catastrophic failures or environmental contamination (e.g., Arosio et al. (2017); Gunn et al. (2018)). Following the example of Gunn et al. (2015), detailed daily reports assessing the level of risk for specific areas of TSFs and WRPs could be generated from TL-ERT measurements to support the operators in charge of stability monitoring. As discussed by Robinson et al. (2008a), TL-ERT could be used to “fill the gap” between point sensor measurements and remote sensing imagery, that are currently the two main strategies developed in EWS monitoring programs for mining wastes (e.g., Li et al. (2020); Lumbroso et al. (2021)).

## Conclusions

Tailing storage facilities (TSF) and waste rock piles (WRP) represent one of the main environmental concerns related to the mining activities. These large scale structures must be properly monitored in order to manage the risk of geotechnical and geochemical instabilities, which can have dramatic consequences. This review discusses the strong potential of long-term time-lapse electrical resistivity tomography (TL-ERT) for the monitoring of key physical properties within mining wastes. Indeed, TL-ERT could fill the gap between conventional point sensors and surface observations, both in terms of spatial and temporal resolution. A systematic review of TL-ERT studies over the last 30 years has been carried out to appraise a state of the art of geoelectrical monitoring and review recent developments, both in terms of types of application and technical advances. The present systematic review of ERT studies applied to mining wastes helps to identify future promising applications of TL-ERT for (i) mining waste valorization, (ii) mining waste characterization, (iii) early detection of contaminated drainage generation, and lastly for (iv) long-term geotechnical and geochemical stability monitoring of TSFs and WRPs. Finally, the most promising perspectives for the future development of TL-ERT monitoring in mining wastes are discussed. In particular, several recommendations concerning field instrumentation, data acquisition and data interpretation are proposed to overcome the challenges that are likely to emerge from a broader use of TL-ERT monitoring in mining wastes.

## Supplementary Materials

Supplementary material for Figure 2 (review and map of mining waste surface in Canada) is available online on the https://adridim.github.io/review2022/0_welcome.html. The database of TL-ERT studies for various types of applications (Sect. 3.1) and the database of ERT studies for mining wastes only (Sect. 4.1) are both available on the as well through maps, graphs and interactive tables that can be downloaded. Databases are also available upon request to the corresponding author.
